# Persistent Solid-State Bipyridine Atropisomerism in a Ferrocenyl-Functionalized Chiral BINOL Scaffold

**DOI:** 10.1021/acs.cgd.6c00771

**Published:** 2026-07-21

**Authors:** Humberto A. Rodríguez, Daniel A. Cruz, Victor Lavin, Juan I. Padrón, Pablo Lorenzo-Luis

**Affiliations:** † Instituto de Productos Naturales y Agrobiología, Consejo Superior de Investigaciones Científicas (IPNA-CSIC), 38206 La Laguna, Tenerife, Islas Canarias, Spain; ‡ Departamento de Física, MALTA-Consolider Team, and IUdEA, Universidad de La Laguna, Apartado de Correos 456, San Cristóbal de La Laguna, Santa Cruz de Tenerife E-38200, Spain; § Área de Química Inorgánica, Departamento de Química, Universidad de La Laguna, C/Astrof?sico Francisco S?nchez 3, 38071 La Laguna, Spain

## Abstract

In this work, we report the synthesis and structural characterization of a novel organometallic bipyridine-based atropisomer featuring two (*S*)-BINOL-derived units, each functionalized with a ferrocene moiety and linked by a 4,4′-disubstituted 2,2′-bipyridine bridge. Although the molecule appears symmetric, single-crystal X-ray diffraction establishes its axial chirality, as demonstrated by Flack *x* = −0.020(14) and Hooft *x* = 0.013(18) parameters. The bipyridine core, sterically hindered by the bulky ferrocenyl groups, imposes a rigid atropisomeric structure. To better understand this behavior, a new Cu­(II) complex is presented to examine how peripheral substitution affects the accessibility and coordination capacity of the bipyridine core; these factors ultimately dictate the solid-state atropisomerism and rigidity of the resulting multinuclear architecture.

## Graphical abstract

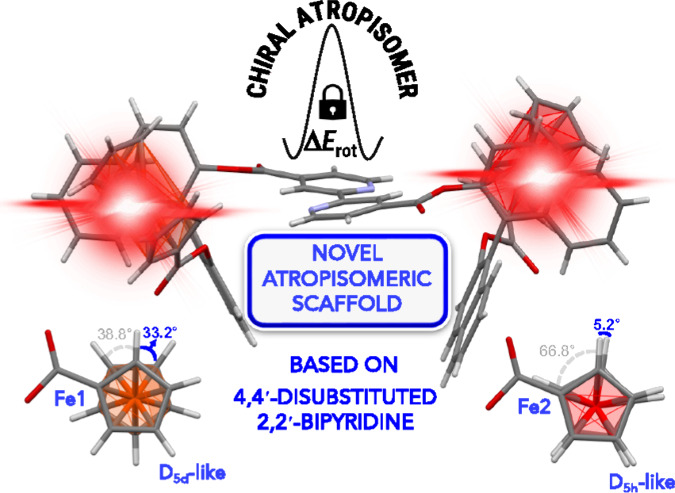

Atropisomerism is a form of axial chirality that arises when rotation about a single bond is hindered by a sufficiently large energy barrier, preventing interconversion and allowing isolation of stable conformers.^1^ This phenomenon is characteristic of biaryl systems bearing bulky ortho substituents and bi-2-napthalene derivatives, for example, 1,1′-bi-2-naphthol (BINOL) which exemplifies axial chirality and serves as a rigid, “privileged” scaffold widely used in asymmetric catalysis.^2^ In addition to classic carbocyclic biaryl scaffolds, 2,2′-bipyridines can also display axial chirality when appropriately substituted, as discussed above. While pristine bipyridine adopts a planar conformation due to delocalized nitrogen lone pairs and electronic repulsion, steric hindrance from bulky groups at the pyridine units can induce a twisted conformation, leading to restricted rotation around the inter-ring bond.

When the rotational barrier is sufficiently high, these systems become configurationally stable and can be isolated as individual enantiomers.^3^ Although this class of atropisomers is less common than their all-carbon analogues, it has garnered increasing attention due to its potential applications in asymmetric catalysis, supramolecular chemistry, and materials science.^4^ Overall, these observations demonstrate that heterocyclic biaryls, when appropriately substituted, can also adopt persistent chiral conformations analogous to those of their carbocyclic counterparts.

Incorporating ferrocenyl units into axially chiral frameworks provides additional structural and functional advantages, as ferrocene offers not only considerable steric bulk but also unique redox and optical properties.^5^ Despite its widespread use in planar-chiral ligands,^6^ its integration into atropisomeric structures remains relatively underexplored, especially in systems involving 4,4′-disubstituted 2,2′-bipyridines and (*S*)-BINOL derivatives (Figure 1).^7^

**Figure 1:**
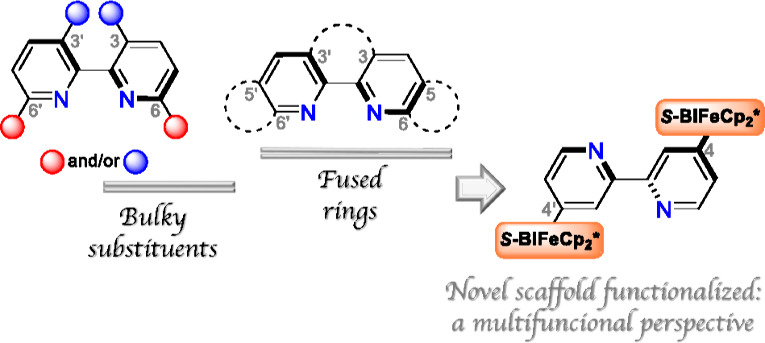
(gray) The most common literature strategies for generating bipyridine atropisomers are shown, based on bulky substituents and ring-fused systems.^7^ (orange) The approach developed in this work is highlighted, featuring a chiral BINOL scaffold functionalized with a ferrocenyl unit at the 4,4′ positions (*S*-BIFeCp_2_*).

On the basis of these considerations, we report the synthesis of the novel BINOL–bipyridine–ferrocene scaffold **5**·EtOAc, in which the bipyridine core exhibits persistent atropisomerism as a consequence of restricted rotation imposed by the bulky BINOL–ferrocenyl substituents. This scaffold is obtained through two consecutive Steglich esterifications,^8^ using DIC and DMAP (Figure 2).

**Figure 2:**
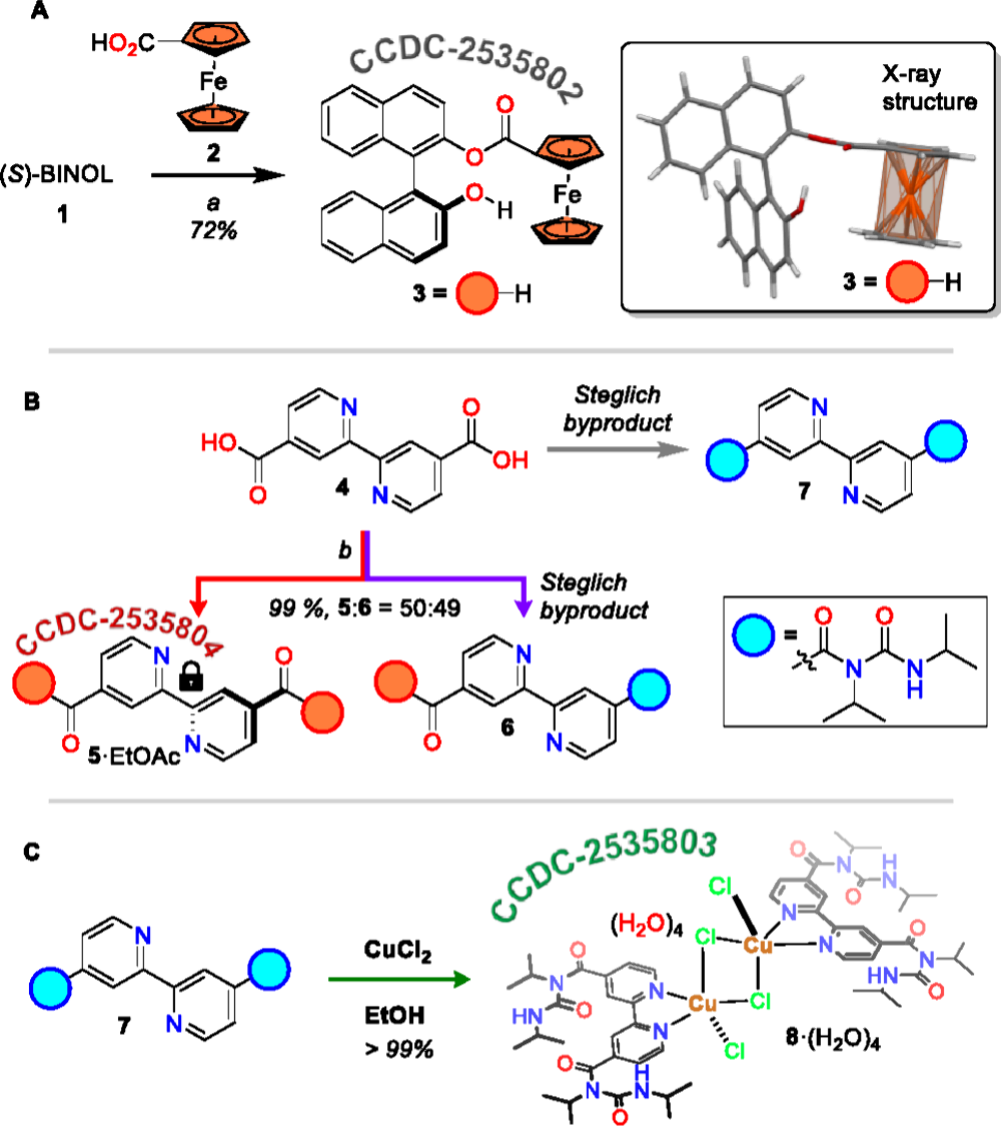
Proposed pathways for the conversions of **1** → **3** (A-top), **4** → **5**, with byproducts **6** and **7** indicated (B-middle), and **7** → **8**·(H_2_O)_4_ (C-bottom). Conditions a: 1.0 equiv of **2**, 1.2 equiv of DIC, 0.3 equiv of DMAP, and 1.0 equiv of 1 in DCM under N_2_ at 25 °C overnight. Conditions b: 5.0 equiv of **4**, 10.0 equiv of DIC, 5.0 equiv of DMAP, and 1.0 equiv of **3** in DCM under N_2_ at reflux overnight. DIC = *N*,*N*′-diisopropylcarbodiimide; DMAP = 4-dimethylaminopyridine.

The first step involves the reaction of (*S*)-BINOL **1** with ferrocenecarboxylic acid **2**, affording precursor and BINOL derivative **3** in 72% yield. Notably, an acylurea was also isolated as a known side product,^9^ arising from a thermodynamically favored pathway that competes with ester formation under these conditions. This competitive pathway became even more pronounced during the second esterification step toward chiral dimer **5·**EtOAc, leading to the isolation of bipyridine derivative **6** in 49% yield.

The overall low selectivity of the system was further attributed to the poor solubility of 2,2′-bipyridine-4,4′-dicarboxylic acid **4** in dichloromethane, which required reflux conditions to observe any detectable reactivity (Table S2 in the Supporting Information).

As shown in Figure 3, the luminescent properties of each of the investigated substrates were examined. Notably, the diffuse reflectance spectrum of atropisomeric **5**·EtOAc can be rationalized as a combination of the spectral features of its individual components. The absorption maximum at 446 nm can be associated with metal-centered d–d transitions (a_1g_ → e_1g_),^10^ while the shoulders observed at 320 and 275 nm are attributed to π–π* transitions of the (*S*)-BINOL **1** moiety and 2,2′-bipyridine-4,4′-dicarboxylic acid **4**,^11^ respectively. In addition, a broad shoulder centered at 576 nm was detected and tentatively assigned to a metal-to-ligand charge transfer (MLCT) band.^12^

**Figure 3:**
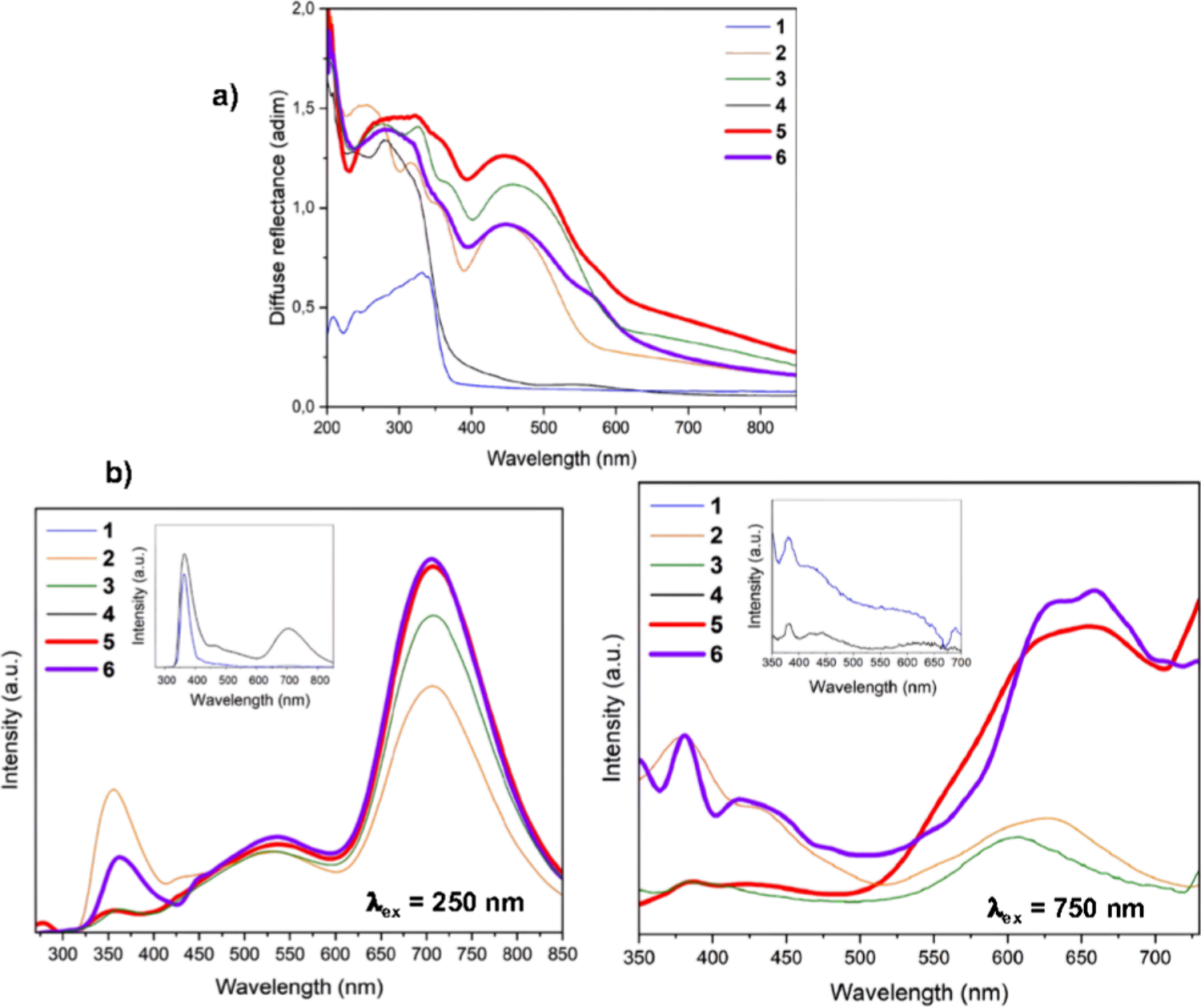
(a) The diffuse reflectance spectra. (b) The emission (left, λ_exc_ = 250 nm) using a xenon lamp and a 395 nm cutoff filter and excitation (right, λem = 750 nm) spectra recorded without optical filters. (a.u. = arbitrary units.)

In the emission spectrum, the ratio of the intensities at 706 nm (deep red) and 358 nm (UV) is remarkably high (15.4:1) for the atropisomer **5**·EtOAc compared with the other compounds investigated in this study. For instance, commercially available ferrocenecarboxylic acid exhibits only a 1.9:1 ratio, highlighting the dramatic enhancement of red luminescence upon incorporation into the atropisomeric **5**·EtOAc. Such an intense red-to-blue emission ratio is appealing for the development of luminescent crystalline materials. The excitation spectra reveal that ferrocene carboxylic acid **2** and BINOL derivative **3**, exhibit maxima at 632 and 600 nm, respectively, which can be attributed to excitation of the FeCp_2_ moieties. In contrast, for chiral dimer **5**·EtOAc and bipyridine derivative **6** the lowest-energy excitation band is red-shifted to about 654 nm (with **6** showing a shoulder at 634 nm), indicating that the bipyridine environment modulates the MLCT transition. This red shift is consistent with literature reports showing that the d–d band of ferrocene can be shifted to longer wavelengths upon functionalization. Here, the bipyridyl environment stabilizes the π* orbitals, leading to a lower-energy MLCT. For the atropisomer **5**·EtOAc, the band at 430 nm is comparatively weak, giving an intensity ratio (654 nm/430 nm) of 15.7:1, further underscoring the dominance of the FeCp_2_ centered transition.

Overall, these findings show that the emission and excitation bands associated with the FeCp_2_ unit are clearly enhanced relative to the organic chromophores of the molecule. The rigid atropisomeric structure likely suppresses nonradiative decay pathways and favors efficient red MLCT emission, while the bipyridine environment contributes to a strong charge-transfer character.^10^ Although compounds **2**, **4**, and **5·**EtOAc exhibit easily detectable luminescence, their quantum yields are very low. In particular, sample **4** shows red emission that is visible to the naked eye under a 250-xenon lamp or a 375 nm cw laser light, using a 610 nm long-pass filter, despite having a low but measurable quantum yield of 0.6% (Figures S24 and S25 in the Supporting Information).

Regarding BINOL derivative **3**, it crystallizes in the monoclinic space group *P*2_1_ (*Z* = 2, *Z*′ = 1). A moderate hydrogen-bond interaction (D–H···A/Å, °: D···A, H···A), O3–H2···O2:2.836(5), 2.113(4), 147.0(3), was observed and appears to contribute to the spatial arrangement of the FeCp_2_ fragment, which is located between the naphthalene units of neighboring molecules (Figure 4a). The Cp rings adopt a quasi-staggered conformation, with an average rotational angle of 26.6° (Figure 4b). This value was calculated as the average of the dihedral angles Ct­(C1–C5)–Ci–Cj–Ct­(C6–C10), where Ct represents the centroid of the Cp ring and Ci/Cj correspond to the respective carbon atoms of each Cp ring, resulting in a D_5d_-like conformation.^13^

**Figure 4:**
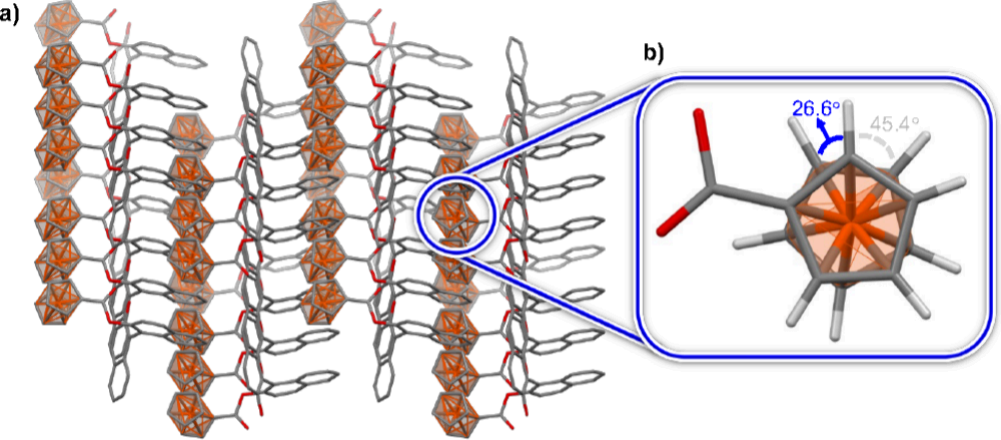
(a) View of **3** along the a* crystallographic axis of the 4 × 2 × 2 (*abc*) supercell, showing the stacking of the FeCp2 units (hydrogen atoms omitted for clarity). (b) Alternating arrangement of the Cp rings. (Atom color code: orange = Fe, gray = C, red = O, white = H.)

The C····C distances between the Cp rings range from 3.30(1) to 3.38(1) Å (average 3.34 Å), while the Fe–C distances for the free Cp ring vary between 2.02(1) and 2.03(1) Å (*mean* 2.03 Å), and between 2.016(6) and 2.055(7) Å (*mean* 2.035 Å) for the second Cp unit. Notably, Fe1 is not equidistant from both Cp rings. The distance from the metal center to the centroid of the Cp ring attached to the binolic unit is 1.6413(6) Å, whereas the distance to the second Cp ring is longer by 0.016(6) Å. This slight asymmetry may arise from partial electronic deactivation of the Cp ring through conjugation with the carbonyl group, shifting the metal center toward that direction.

In contrast, **5**·EtOAc crystallizes in the triclinic space group *P*1 (*Z* = *Z*′ = 1, Table S9 in the Supporting Information), with the absolute configuration shown in Figure 5a. Flack *x* = −0.020(14) and Hooft *x* = 0.013(18) parameters, unequivocally support the correct assignment of the absolute configuration, ruling out racemization during synthesis.^14^ A value close to 0.5 would indicate the presence of a racemic mixture, which is not observed here. This assignment is consistent with the formation of a quasi-*anti* atropisomer with restricted rotation.^15^ Optical rotation measurements {[α]_D_^20^ = −4.8; *c* = 5.03, CHCl_3_} further show that dimer is optically active in solution. The crystal structure exhibits an ABAB-type packing arrangement of chiral dimer stacked with an ethyl acetate molecule, with slight positional disorder and was addressed by refining its atomic occupancy factors (0.6/0.4) (Figure 6). One face of the structure is strongly hindered by the naphthalene substituents. The *inter*centroid distance between the py1 ring (N1–C39–C37–C40–C38–C41) and the py2 ring (N2–C35–C34–C32–C33–C36) is 7.7(7) Å along an axis (Figure 5b), indicating the absence of π–π interactions between the bipyridine units.^16^ The structural relevance of this compound lies in the orientation of the pyridine rings, which are not coplanar; the N2–C36–C37–N1 torsion angle is −172.6(4)°.

**Figure 5:**
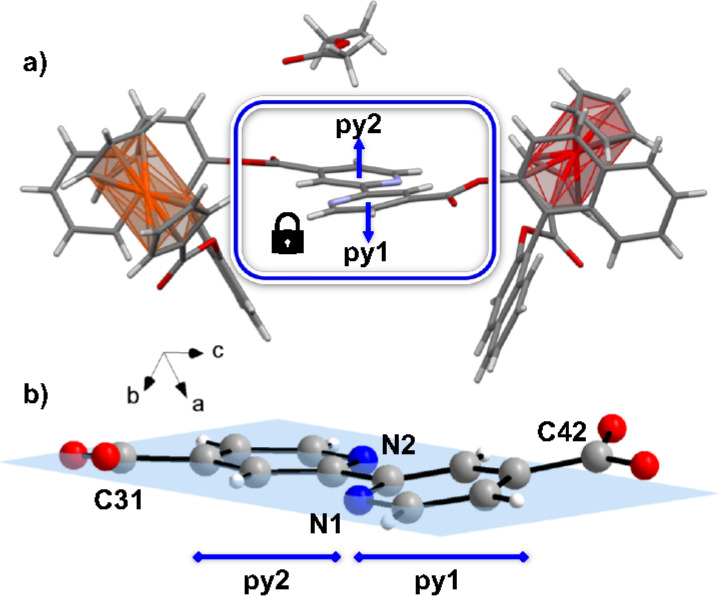
(a) Molecular structure of **5·**EtOAc, highlighting the pyridine units of the bipyridine framework in the restricted configuration. Two colors are used to distinguish the ferrocenyl derivatives. (b) Plane defined by the atoms of the py2 unit.

**Figure 6:**
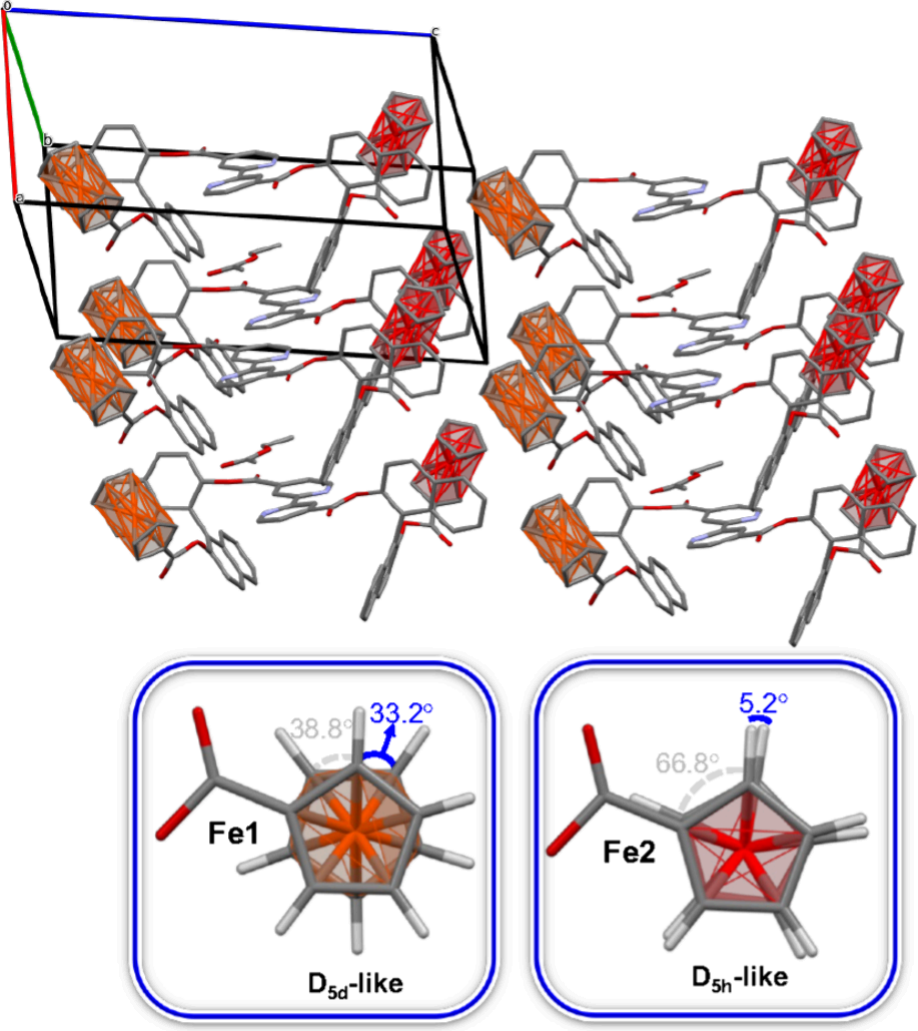
(top) Packing diagram of **5**·EtOAc showing ABAB stacking along the crystallographic axis (hydrogen atoms omitted for clarity). (bottom) The panels show the symmetry-related fragments around Fe1 and Fe2, together with the average dihedral angles between the Cp rings and the attached carbon atoms.

The least-squares plane associated with the py2 unit was calculated, and the distances of selected atoms belonging to py1 and the adjacent ester groups from this plane were determined (Table 1), revealing that none of them are perfectly aligned with the defined plane.This arrangement is supported by computational studies on substituted 2,2′-bipyridines, which indicate that substitution at the 4,4′ positions destabilize the *syn* conformer through increased steric interactions with the 3,3′-hydrogen atoms, thereby favoring the *anti*-geometry and increasing the rotational barrier.^17^ Consistent with this, the fragment containing Fe1 displays a staggered conformation with an averaged torsion angle Ct­(C39–C41–C42–C48–C49)–Ci–Cj–Ct­(C43–C47) of 33.2°, corresponding to a D_5d_-like conformation, which is more symmetric than that observed for BINOL derivative **3**. In contrast, the fragment containing Fe2 adopts a quasi-eclipsed conformation, Ct­(C14–C18)–Ci–Cj–Ct­(C69–C73) = 5.2°, corresponding to a D_5h_-like arrangement.

**Table 1:** Distances of Selected Atoms from the Plane Are Defined by the py2 Unit

Atom	*d* (Å)	Atom	*d* (Å)
N1^a^	–0.110(4)	C31	0.049(5)
C37^a^	0.047(5)	O3	0.268(4)
C38^a^	0.283(5)	O4	–0.070(4)
C39^a^	–0.0547(6)	C42	0.681(6)
C40^a^	0.182(6)	05	0.981(4)
C41^a^	0.370(5)	06	0.683(4)

a Atoms belonging to the py1 unit.

These conformations may be influenced by weak interactions within the crystal lattice. For the fragment containing Fe1, intramolecular C–H···π interactions involving the centroids of the naphthalene rings (Cg_*n*_) were identified (C–H···Cg_*n*_ (Å, °): C···Cg_*n*_, H···Cg_*n*_): C3–H3···Cg1 (C21–C22–C27–C28–C29-C30): 3.479(6), 2.65(6), 148.0(7) and C4–H4···Cg2 (C14–C15–C16–C17–C18-C19): 3.737(7), 2.87(7), 156.0(7).^18^ Additionally, the fragment containing Fe2 exhibits a intermolecular interaction of the same type (C–H···Cg_*n*_ (Å,°): C···Cg_*n*_, H···Cg_*n*_): C67–H67···Cg3 (C46–C47–C48–C49–C50-C51): 3.605(6), 2.732(5), 156.5(7) with a neighboring unit associated with Fe2. Such weak *intra*- and *inter*-molecular interactions contribute to the stabilization of the solid-state arrangement. Thermal stability studies confirm this structural robustness: while bipyridine derivative **6** decomposes gradually upon heating, atropisomer **5·**EtOAc remains stable up to 300 °C. This superior stability of **5**, after the loss of the ethyl acetate molecule, is likely due to the stabilizing effect of the additional ferrocenyl moiety (Figures S2 and S3 in the Supporting Information).

To establish a direct structure–function comparison within this family of 2,2′-bipyridine derivatives, we next examined the coordination behavior of the Steglich-derived bipyridine byproduct **7** toward CuCl_2_. Compound **7**, bearing two dicarboxamide arms and displaying low solubility in a wide range of solvents (Section S1.2 in the Supporting Information), provides a useful reference platform to assess how peripheral substitution affects the coordination ability of the bipyridine core. This comparison is particularly relevant because the ferrocenyl-functionalized scaffold **5**·EtOAc exhibits a sterically congested bipyridine environment, whereas **7** retains sufficient accessibility at the nitrogen donors to engage in metal coordination.

Upon suspending **7** in ethanol and adding an ethanolic solution of CuCl_2_ at room temperature, the complex **8·**(H_2_O)_4_ could be isolated in near-quantitative yield (99%) (Figure 2). This observation highlights that the ferrocenyl moieties present in **5**·EtOAc and bipyridine derivative **6** significantly reduce the coordinating ability of the bipyridine core, preventing the formation of novel bimetallic complexes.

Complex **8·**(H_2_O)_4_ crystallizes in the monoclinic space group *P*2_1_/*n* (*Z* = 2, *Z*′ = 0.5) with excellent crystallographic quality parameters (Table S10, Supporting Information). A notable structural feature is the formation of two μ-Cl bridges between the Cu­(II) centers, despite the stoichiometric metal-to-ligand ratio employed during the synthesis. The Cu–Cl distances are significantly different: the distances Cu1–Cl1 and Cu1–Cl2 are 2.2608(7) and 2.499(9) Å, respectively, whereas the distance Cu1–Cl1′ is 2.6275(6) Å, which is remarkably longer, as reported in related systems.^19^ The angle Cu1–Cl1–Cu1 is 91.46(2)°, while the bite angle of the bipyridine ligand, N1–Cu1–N2, is 79.66(7)°. The Addison parameter (τ_5_) was calculated to be 0.49,^20^ considering the angles β (N2–Cu1–Cl1) = 173.16(6)° and α (N1–Cu1–Cl2) = 147.01(6)°. This value indicates a distorted geometry almost perfectly intermediate between a square-pyramidal and a trigonal-bipyramidal (τ_5_ = 0 and 1, respectively).

This coordination geometry mainly arises from the structural distortion imposed by the bipyridine moiety and the strain associated with the μ-Cl bridges within the metal coordination sphere. Regarding the organic framework, it is noteworthy that the adopted conformation orients the carboxamide groups of the bipyridine unit along the same axial direction (with the symmetry-related fragment oriented oppositely). Structural analysis reveals π···π stacking interactions are observed with an interplanar distance of 3.6708(1) Å,^17^ together with several hydrogen-bonding interactions involving solvated water molecules and the carboxamide arms (Figure 7).

**Figure 7:**
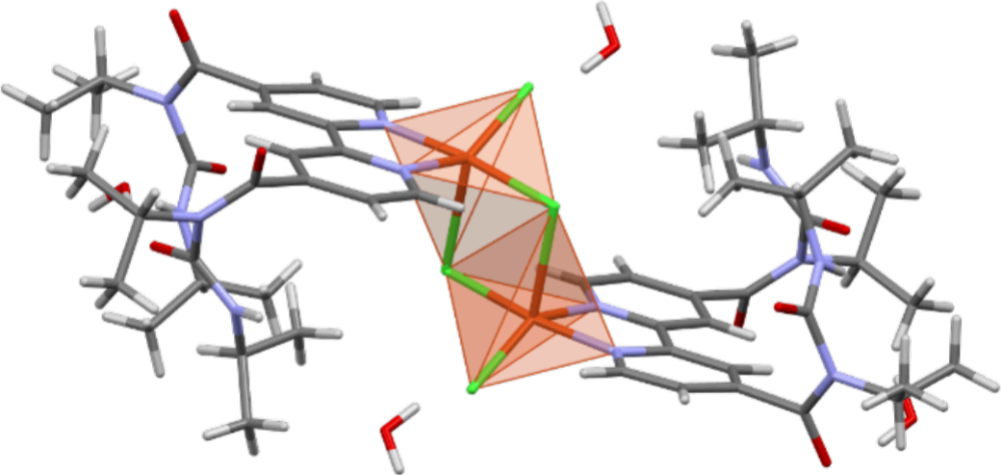
Crystal packing of **8·**(H_2_O)_4_. The noncovalent interactions omitted for clarity.

These include interactions of the type [D–H···A (Å,°): D···A, H···A): O1W–H1Wa···O2:2.885(4), 2.04(2), 168.3(1) and O2W–H2Wa···O3:4.991(4), 4.23(4), 151.2(4)], as well as interactions between the water molecules themselves: O2W–H2 Wb···O1W: 2.803(5), 2.05(3), 146.1(3).

It should be noted that the amide groups participate in further hydrogen-bonding interactions with both solvated water molecules and chlorine atoms that are not involved in the Cu–Cl bridges: N6–H6···O1W: 2.926(4), 2.075(4), 170.0(3)° and Cl2···O2W: 3.196(4) Å, respectively. This extensive network of noncovalent interactions favors a preferred spatial arrangement in which the carboxamide arms are approximately aligned within the same plane. Furthermore, a hydrogen bonding between carboxamide groups belonging to different units further reinforces the supramolecular network in the framework [(D–H···A (Å,°): D···A, H···A): N4–H4···O4:2.968(3), 2.113(3), 172.7(3)].

This example highlights the remarkable relevance of carboxamide functionalities in complex formation, because of their ability to engage in multiple hydrogen-bonding interactions that contribute significantly to the structural stabilization of the solid-state framework. Such interactions are widely exploited in supramolecular chemistry and organocatalysis.^21^ Finally, thermal degradation profiles align with the theoretical predictions for the scaffold’s intrinsic stability (Figure S4 in the Supporting Information). This process does not interfere with the overall structural analysis, as evidenced by the detailed characterization included in the Supporting Information.

In summary, a novel BINOL–bipyridine–ferrocene scaffold exhibiting persistent atropisomerism has been successfully developed and structurally characterized. The steric impact of BINOL-ferrocenyl moieties effectively locks the bipyridine unit, ensuring configurational stability, while simultaneously reducing its coordination ability due to electronic deactivation. Photophysical studies confirm that compounds **2**, **4**, and **5·**EtOAc are luminescent but exhibit very low emission efficiencies, with quantum yields remaining below 1%.

While the sterically congested ferrocenyl-containing scaffolds **5**·EtOAc and bipyridine derivative **6** show reduced metal-binding ability, the related Steglich-derived bipyridine derivative **7** retains sufficient accessibility at the nitrogen donors to afford the dinuclear Cu­(II) complex **8**·(H_2_O)_4_, which is stabilized by an extensive network of hydrogen-bonding and π-interactions.

Overall, these results demonstrate how subtle structural modifications in bipyridine-based systems can govern the interplay between atropisomerism, coordination properties, and supramolecular organization, providing useful insights for the design of functional materials in crystal engineering.

## Supplementary Material



## Notes

Detailed synthetic procedures, characterizations, and single-crystal X-ray crystallographic data (PDF)
