# Supplementary material for: Persistent Solid-State Bipyridine Atropisomerism in a Ferrocenyl-Functionalized Chiral BINOL Scaffold
Source: Cryst Growth Des. 2026 Jul 21;26(15):5738–43. doi: 10.1021/acs.cgd.6c00771 (PMC13449761; doi:10.1021/acs.cgd.6c00771)
Supplement: Supplementary file 1 [file cg6c00771_si_001.pdf]

Supplementary Information

Persistent Solid-State Bipyridine Atropisomerism in a Ferrocenyl-Functionalized Chiral BINOL Scaffold

Humberto A. Rodríguez,[a, b] Daniel A. Cruz,[a] Victor Lavin,[c] Juan I. Padrón[a*] and Pablo Lorenzo-Luis[d*]

[a] Instituto de Productos Naturales y Agrobiología, Consejo Superior de Investigaciones Científicas (IPNA-CSIC), 38206, La Laguna, Tenerife, Islas Canarias: Email: jipadron@ipna.csic.es

[b] Área de Química Inorgánica, Departamento de Química, Universidad de La Laguna, C/ Astrofísico Francisco Sánchez 3, 38071 La Laguna, Spain. Email: plorenzo@ull.es

[c] Departamento de Física, MALTA-Consolider Team, and IUdEA, Universidad de La Laguna, Apartado de Correos 456, San Cristóbal de La Laguna, Santa Cruz de Tenerife E-38200, Spain. Email: vlavin@ull.es

Table of Contents

1. Material, experimental procedures and compound data ... 2
1.1. Material ... 2
1.2. Experimental procedure and characterization of ligands ... 3
1.3. Experimental procedure and characterization of **8**·$(H_2O)_4$ ... 5
1.4. Metal scope for **3** ... 5
1.5. Reaction optimization for the synthesis of **5**·EtOAc, **6** and **7** ... 6
1.6. Metal scope for **5**·EtOAc ... 6
1.7. Metal scope for **6** ... 7
1.8. Metal scope for **7** ... 7
2. NMR spectra ... 8
3. Infrared analysis of 8·$(H_2O)_4$ ... 12
4. Thermal Analysis ... 13
5. ESI-MS measurements of 8·$(H_2O)_4$ ... 14
6. Optical characterization ... 15
6.1. (*S*)-BINOL (**1**) ... 15
6.2. Ferrocenecarboxylic acid (**2**) ... 16
6.3. Ferrocene, [[[(*S*)-(2'-hydroxy-[1,1'-binaphthalen]-2-yl]oxy]carbonyl]- (**3**) ... 17
6.4. 2,2'-bipyridine-4,4'-dicarboxylic acid (**4**) ... 18
6.5. Bis((*S*)-2'-((ferrocenecarbonyl)oxy)-[1,1'-binaphthalen]-2-yl)[2,2'-bipyridine]-4,4'-dicarboxylate (**5**).. 19
6.6. (*S*)-2'-((ferrocenecarbonyl)oxy)-[1,1'-binaphthalen]-2-yl 4'-(isopropyl(isopropylcarbamoyl)carbamoyl)-[2,2'-bipyridine]-4-carboxylate (**6**) ... 20
6.7. Photographic evidence of visible red emission from **4** ... 21
7. X-Ray diffraction data ... 22
7.1. Crystal information of **3** ... 23
7.2. Crystal information of **5**·EtOAc ... 24
7.3. Crystal information of **8**·$(H_2O)_4$ ... 25

1. Material, experimental procedures and compound data

1.1. Material

NMR spectra were recorded on a **Bruker Advance instrument**. ^{1}H NMR spectra were recorded at 400 and 500 MHz, and ^{13}C NMR were recorded at 100 and 125 MHz, VTU 298.0 °K. Chemical shifts were reported in parts per million. The residual solvent peak was used as an internal reference ($CDCl_3$: δ_H 7.26, δ_C 77.0).

Optical rotations were measured on an **Anton Paar- M150 Polarimeter**. **HRESMS** data were performed on a **Waters SYNAPT XS** ion mobility **Q-TOF** mass spectrometer by addition of CHO_2H (10% in water). **Infrared spectra** were measured in **FTIR-Cary 630 Spectrometer** of Agilent. Determination of the **melting point** with the instrument **M-560 Büchi. Diffuse reflectance** spectra were made using a spectrophotometer equipped with an integrating sphere (**Cary 5000**). **Emission and excitation** spectra were measured using a photoluminescence spectrometer (**Edinburgh Instrument FLS1000**).

For analytical **thin-layer chromatography**, silica gel ready-foils were used, respectively, being developed with 254 nm UV light and/or spraying with a solution of phosphomolybdic acid solution (20 wt. % in ethanol) or vainillin stainand and heating. **Column chromatography** was performed using silica gel (0.015-0.04 mm) and *n*-hexane/EtOAc solvent system with **Biotage Isolera®** system. **Recycling HPLC** was performed on a **LaboACE LC-5060 Plus II** (**JAI**) equipped with a JAIGEL-HR column using $CHCl_3$ as the eluent to purify some products. All reagents were obtained from commercial sources (Sigma-Aldrich®, Apollo Scientific®, Thermo Scientific®, TCI Chemicals® and BLDpharm®) and used without any further purification. Dry solvents were used from the **PureSolv system**.

The reagents used have been obtained from the following **commercial sources**, indicating the purity of each of them. The following reagents were purchased from the commercial company **Sigma-Aldrich®**: *N*,*N*'-diisopropylcarbodiimide 99% (CAS No. 693-13-0), copper(II) chloride 99.9% (CAS No. 7447-39-4), tetrakis(acetonitrile)copper(I) hexafluorophosphate 97% (CAS No. 4443-05-6), lanthanum(III) trifluoromethanesulfonate 99.999% (CAS No. 52093-26-2), iron(III) trifluoromethanesulfonate 90% (CAS No. 63295-48-7), bis(acetonitrile)dichloropalladium(II) 99% (CAS No. 14592-56-4) and iron(III) chloride 98% (CAS No. 7705-08-0). The following reagents were purchased from the commercial company **Apollo Scientific®**: (*S*)-(-)-1,1'-bi(2-naphthol) 99% (CAS No. 18531-99-2), 2,2'-bipyridine-4,4'-dicarboxylic acid 98% (CAS No. 6813-38-3), *N*,*N*-dimethylpyridi*n*-4-amine 98% (CAS No. 1122-58-3), copper(II) trifluoromethanesulphonate 98% (CAS No. 34946-82-2), palladium(II) acetate 99.5% (CAS No. 3375-31-3) and ytterbium(III) trifluoromethanesulphonate 97% (CAS No. 54761-04-5) . The following reagents were purchased from the commercial company **Thermo Scientific®**: Ferrocenecarboxylic acid 97% (CAS No. 1271-42-7), copper(II) bromide 99% (CAS No. 7789-45-9) and terbium(III) trifluoromethanesulfonate 98% (CAS No. 148980-31-8). The following reagents were purchased from the commercial company **TCI Chemicals®**: Copper(I) iodide 98% (CAS No. 7681-65-4). The following reagents were purchased from the commercial company **BLDpharm®**: Indium(III) trifluoromethanesulfonate 99% (CAS No. 128008-30-0) and 1,4-dioxane 99% (CAS No. 123-91-1).

1.2. Experimental procedure and characterization of ligands

1.2.1. Ferrocene, [[[(*S*)-(2'-hydroxy-[1,1'-binaphthalen]-2-yl]oxy]carbonyl]⁻ (3) and Ferrocene, [[(1-methylethyl)[[(1-methylethyl)amino]carbonyl]amino]carbonyl]⁻ (3b)

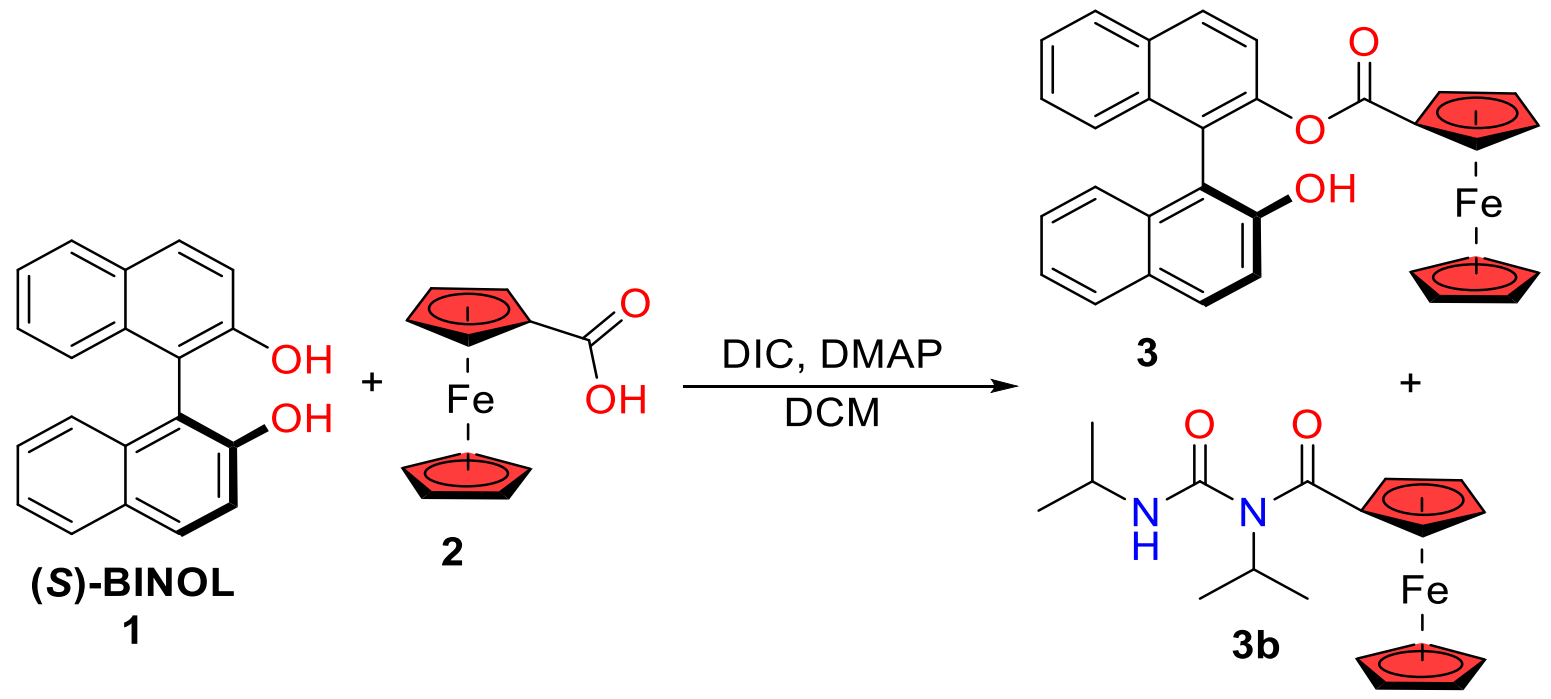

To a solution of ferrocenecarboxylic acid **2** (123.0 mg, 0.52 mmol, 1.0 equiv) in dry DCM (0.1 M), under nitrogen at 25°C were sequentially added *N*,*N'*-diisopropylmethanediimine (0.10 mL, 0.62 mmol, 1.2 equiv) and *N*,*N*-dimethylpyridin-4-amine (19.4 mg, 0.16 mmol, 0.3 equiv). After 1 h of vigorous stirring, (*S*)-BINOL **1** (150.0 mg, 0.52 mmol, 1.0 equiv) is added, stirring overnight at 25°C. Then HCl 1M solution was added and the reaction mixture was extracted with DCM, the organic phases were mixed and dried with $MgSO_4$. The crude reaction mixture was purified using a Biotage Isolera (silica gel, isocratic start in 95:5 *n*-Hex/EtOAc then 95:5 *n*-Hex/EtOAc to 60:40 *n*-Hex/EtOAc gradient) to provide 187.2 mg (0.38 mmol, **72%**) of **3** as orange solid and traces of **3b**. Spectroscopic data of **3b** matches with those reported in literature.[1] The crystallization of **3** was carried out in 3:1 EtOAc/DCM at room temperature (red cubes).

3: ^{1}H NMR (400 MHz, $CDCl_3$) δ 8.02 (d, *J* = 8.9 Hz, 1H), 7.93 (d, *J* = 8.2 Hz, 1H), 7.87 (d, *J* = 8.9 Hz, 1H), 7.82 – 7.77 (m, 1H), 7.46 (t, 1H), 7.42 – 7.35 (m, 2H), 7.33 – 7.27 (m, 3H), 7.22 – 7.15 (m, 2H), 5.65 (bs, 1H), 4.64 (bs, 1H), 4.53 (bs, 1H), 4.28 (bs, 1H), 4.25 (bs, 1H), 3.59 (bs, 5H).**^{13}C NMR (100 MHz, $CDCl_3$)** δ 171.7 (C), 152.1 (C), 148.3 (C), 133.8 (C), 133.7 (C), 132.3 (C), 130.9 (CH), 130.6 (CH), 129.3 (C), 128.4 (CH), 128.3 (CH), 127.5 (CH), 126.9 (CH), 126.3 (CH), 125.8 (CH), 124.9 (CH), 123.7 (CH), 123.4 (C), 122.1 (CH), 118.7 (CH), 114.9 (C), 71.9 (CH), 71.8 (CH), 70.6 (CH), 70.5 (CH), 69.8 (5xCH), 69.5 (C). **HRMS (ESI$^+$):** *m/z* $[M-H]^+$ calcd. for $C_{31}H_{21}FeO_3^+$: 497.0829; found: 497.0840. **Active bands in IR (cm^{-1}):** 3387, 3063, 1726, 1696, 1272, 1208, 1101, 816, 749. **$[\alpha]_D^{20}$:** -39.1 (*c* = 1.00, $CHCl_3$). **Melting point:** Decomposes at 214.2°C. See spectrum.

3b: ^{1}H NMR (400 MHz, $CDCl_3$) δ 6.44 (s, 1H), 4.76 – 4.71 (m, 2H), 4.71 – 4.65 (m, 1H), 4.40 – 4.35 (m, 2H), 4.23 (s, 5H), 3.95 – 3.82 (m, *J* = 6.8 Hz, 1H), 1.45 (d, *J* = 6.8 Hz, 6H), 1.06 (d, *J* = 6.6 Hz, 6H). **^{13}C NMR (100 MHz, $CDCl_3$)** δ 172.9 (C), 154.9 (C), 78.3 (C), 70.5 (5xCH), 70.4 (4xCH), 49.5 (CH), 43.0 (CH), 22.6 (2xCH_3), 21.4 (2xCH_3). **HRMS (ESI$^+$):** *m/z* $[M+Na]^+$ calcd. for $C_{18}H_{24}FeN_2O_2Na^+$: 379.1074; found: 379.1085. **Active bands in IR (cm^{-1}):** 3280, 3089, 2969, 2872, 1695, 1614, 1531, 1384, 1311, 1256, 819. See spectrum.

[1] Schetter, B.; Speiser, B. *J. Organomet. Chem.* **2004**, *689* (9), 1472–1480.

1.2.2. bis((*S*)-2'-((ferrocenecarbonyl)oxy)-[1,1'-binaphthalen]-2-yl) [2,2'-bipyridine]-4,4'-dicarboxylate (5), (*S*)-2'-((ferrocenecarbonyl)oxy)-[1,1'-binaphthalen]-2-yl 4'-(isopropyl(isopropylcarbamoyl)carbamoyl)-[2,2'-bipyridine]-4-carboxylate (6) and N^4,$N^{4'}$-diisopropyl-N^4,$N^{4'}$-bis(isopropylcarbamoyl)-[2,2'-bipyridine]-4,4'-dicarboxamide (7)

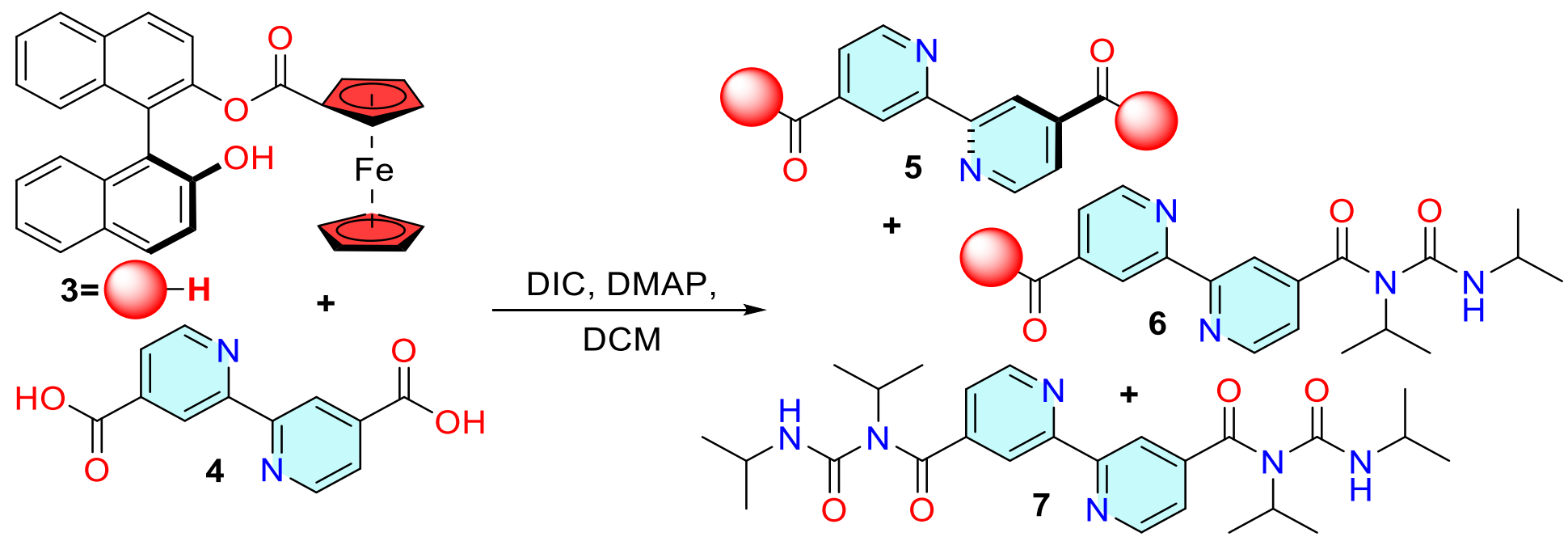

To a solution of [2,2'-bipyridine]-4,4'-dicarboxylic acid **4** (741.0 mg, 3.00 mmol, 5.0 equiv) in dry DCM (0.1 M), under nitrogen at 25°C were sequentially added *N*,*N'*-diisopropylmethanediimine (0.94 mL, 6.01 mmol, 10.0 equiv) and *N*,*N*-dimethylpyridin-4-amine (374.5 mg, 3.00 mmol, 5.0 equiv). After 1 h of vigorous stirring, **3** (300.0 mg, 0.60 mmol, 1.0 equiv) is added, stirring overnight at reflux. Then HCl 1M solution was added and the reaction mixture was extracted with DCM, the organic phases were mixed and dried with $MgSO_4$. The crude reaction mixture was purified using a Biotage Isolera (silica gel, isocratic start in 95:5 *n*-Hex/EtOAc then 95:5 *n*-Hex/EtOAc to 60:40 *n*-Hex/EtOAc gradient) to provide 173.4 mg of **5** (0.15 mmol, 50%) as orange solid, 233.1 mg of **6** (0.29 mmol, 49%) as orange solid and 253.3 mg of **7** (0.51 mmol, 17% respect to **4**) as white solid. The crystallization of **5** was carried out in EtOAc at low temperature (red cubes).

5: ^{1}H NMR (400 MHz, $CDCl_3$) δ 8.58 (d, *J* = 4.9 Hz, 2H), 8.49 (s, 2H), 8.10 (d, *J* = 8.9 Hz, 2H), 7.98 (d, *J* = 8.2 Hz, 2H), 7.87 (dd, *J* = 13.7, 8.7 Hz, 4H), 7.71 (d, *J* = 8.9 Hz, 2H), 7.55 – 7.48 (m, 4H), 7.45 – 7.38 (m, 6H), 7.37 – 7.28 (m, 6H), 4.69 – 4.64 (m, 2H), 4.59 – 4.54 (m, 2H), 4.32 – 4.26 (m, 4H), 3.61 (s, 10H). **^{13}C NMR (125 MHz, $CDCl_3$)** δ 170.6 (2xC), 163.7 (2xC), 156.6 (2xC), 150.3 (2xCH), 147.4 (2xC), 147.0 (2xC), 138.0 (2xC), 133.9 (2xC), 133.7 (2xC), 132.3 (2xC), 131.8 (2xC), 130.2 (2xCH), 130.2 (2xCH), 128.6 (2xCH), 128.5 (2xCH), 127.6 (2xCH), 127.3 (2xCH), 126.8 (2xCH), 126.6 (2xCH), 126.2 (2xCH), 126.0 (2xCH), 124.6 (2xC), 123.3 (2xCH), 123.2 (2xC), 122.2 (2xCH), 121.9 (2xCH), 121.1 (2xCH), 72.0 (2xCH), 71.9 (2xCH), 70.8 (2xCH), 70.7 (2xCH), 70.2 (2xC), 69.9 (10xCH). **HRMS (ESI$^+$):** *m/z* $[M+H]^+$ calcd. for $C_{74}H_{49}Fe_2N_2O_8^+$: 1203.2234; found: 1203.2214. **Active bands in IR (cm^{-1}):** 3064, 2924, 1733, 1363, 1267, 1223, 1205, 1100, 755. **$[\alpha]_D^{20}$:** -4.8 (*c* = 5.03, $CHCl_3$). **Melting point (°C):** 154.7 - 154.9. See spectrum.

6: ^{1}H NMR (500 MHz, $CDCl_3$) δ 8.73 (s, 1H), 8.65 – 8.56 (m, 2H), 8.38 (s, 1H), 8.12 (d, *J* = 8.9 Hz, 1H), 7.99 (d, *J* = 8.1 Hz, 1H), 7.93 (d, *J* = 8.9 Hz, 1H), 7.88 (d, *J* = 8.1 Hz, 1H), 7.73 (d, *J* = 8.9 Hz, 1H), 7.56 – 7.50 (m, 2H), 7.45 – 7.27 (m, 7H), 7.08 – 6.98 (bs, 1H), 4.67 (s, 1H), 4.55 (s, 1H), 4.32 – 4.27 (m, 3H), 3.91 – 3.83 (m, 1H), 3.61 (s, 5H), 1.46 – 1.41 (m, 6H), 1.03 (d, *J* = 6.6 Hz, 3H), 1.00 (d, *J* = 6.5 Hz, 3H). **^{13}C NMR (125 MHz, $CDCl_3$)** δ 170.4 (C), 170.1 (C), 163.2 (C), 156.2 (C), 156.0 (C), 153.3 (C), 150.2 (CH), 150.0 (CH), 147.1 (C), 146.7 (C), 145.5 (C), 137.9 (C), 133.6 (C), 133.4 (C), 132.1 (C), 131.5 (C), 129.9 (CH), 129.9 (CH), 128.4 (CH), 128.2 (CH), 127.3 (CH), 127.0 (CH), 126.5 (CH), 126.4 (CH), 125.9 (CH), 125.8 (CH), 124.3 (C), 123.3 (CH), 123.0 (C), 121.9 (CH), 121.6 (CH), 120.9 (CH), 120.3 (CH), 117.9 (CH), 71.7 (CH), 71.7 (CH), 70.5 (CH), 70.3 (CH), 69.9 (C), 69.6 (5xCH), 50.6 (CH), 43.0 (CH), 22.3 (CH_3), 22.3 (CH_3), 21.0 (CH_3), 20.9 (CH_3). **HRMS (ESI$^+$):** *m/z* $[M]^+$ calcd. for $C_{50}H_{42}FeN_4O_6^+$: 850.2454; found: 850.2488. **Active bands in IR (cm^{-1}):** 3322, 3013, 2972, 2356, 1733, 1701, 1647, 1364, 1222, 1204, 1098, 750. **$[\alpha]_D^{20}$:** -13.8 (*c* = 1.0, $CHCl_3$). **Melting point (°C):** Decomposes at 103.8°C. See spectrum.

7: This compound is highly insoluble, the following deuterated solvents were tested: CD_2Cl_2, $CDCl_3$, C_6D_6, D_2O, MeOD-d$_4$ and DMSO-d$_6$. **HRMS (ESI$^+$):** *m/z* [M]$^+$ calcd. for $C_{26}H_{37}N_6O_4^+$: 497.2876; found: 497.2885. **Active bands in IR (cm^{-1}):** 3301, 2969, 2875, 1705, 1623, 1522, 1383, 1248, 1159. **Melting point (°C):** 191.7 -191.9 °C.

1.3. Experimental procedure and characterization of 8·$(H_2O)_4$

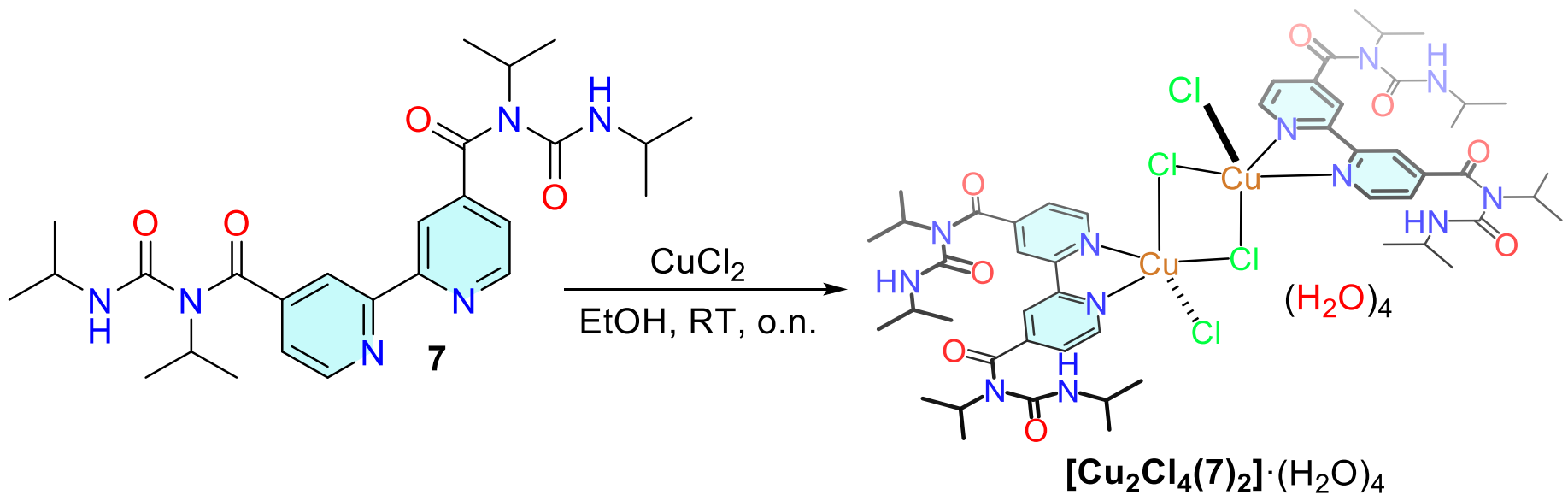

A 0.02 M solution of *N*^{4},*N*$^{4'}$-diisopropyl-*N*^{4},*N*$^{4'}$-*bis*(isopropylcarbamoyl)-[2,2'-bipyridine]-4,4'-dicarboxamide (**7**) (12.9 mg 26.0 µmol, 1.0 equiv) in ethanol was prepared at room temperature. To this, a 0.02 M solution of **$CuCl_2$** (3.5 mg, 26.0 µmol, 1.0 equiv) in ethanol was added slowly, resulting in a color change from cyan to green. The mixture was stirred overnight, after which it was allowed to crystallize slowly at room temperature, obtaining green crystals that were dried in a desiccator, affording **$[Cu_2Cl_4(L)_2]\cdot(H_2O)_4$** in >**99% yield** (9.1 mg, 12.9 µmol).

1.4. Metal scope for 3

Table S1. Coordination conditions tested for **3** with different metals.

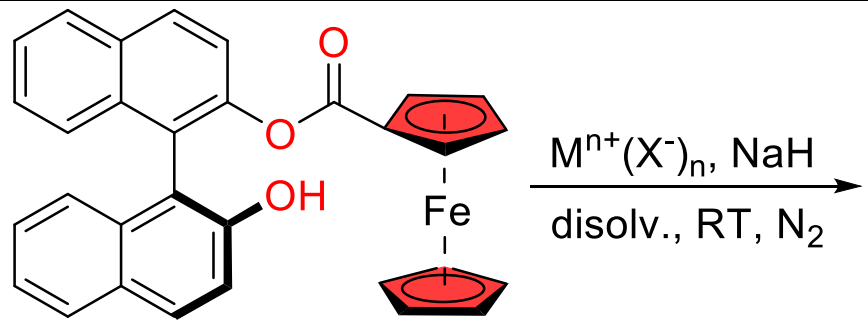

Entry[a]	$M^{n+}(X^-)_n$ (1.0 equiv.)	Solvent (0.05 M)	NaH[b] (equiv.)	Colour	Y[c] (%)
1	$[Cu(ACN)_4]PF_6$	CAN	-	Orange	-
2	$[Cu(ACN)_4]PF_6$	CAN	1.0	Orange	-
3	CuI	CAN	-	Orange	-
4	CuI	CAN	1.0	Orange	-
5	$CuCl_2$	1,4-dioxane	-	Orange	-
6	$CuCl_2$	1,4-dioxane	1.0	Orange	-
7	$CuBr_2$	1,4-dioxane	-	Brown	-
8	$CuBr_2$	1,4-dioxane	1.0	Brown	-
9	$Cu(OTf)_2$	1,4-dioxane	-	Dark orange	-
10	$Cu(OTf)_2$	1,4-dioxane	1.0	Brown	-
11	$Fe(OTf)_3$	1,4-dioxane	-	Dark green	-
12	$In(OTf)_3$	1,4-dioxane	-	Orange	-
13	$La(OTf)_3$	1,4-dioxane	-	Pale orange	-

[a] Reactions were carried out at room temperature under N_2 atmosphere overnight.
[b] Reactions with NaH led to the formation of a precipitate that was insoluble in all solvents tested and was removed from the supernatant.
[c] The crystals that formed corresponded to **3 (CCDC-2535802).**

1.5. Reaction optimization for the synthesis of 5·EtOAc, 6 and 7

Table S2. Reaction condition optimization for the synthesis of **5**·EtOAc, **6** and **7**.

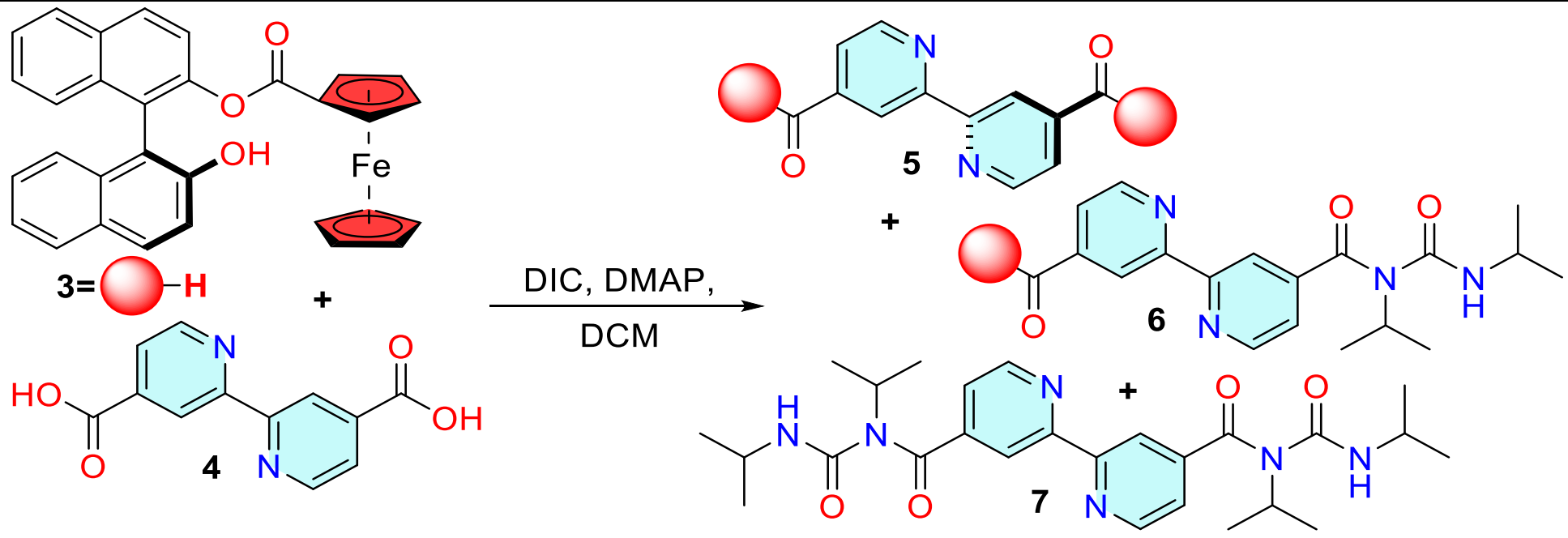

Entry[a]	3 (equiv.)	4 (equiv.)	DIC/DMAP (equiv.)	Solvent[b]	T (° C)	Y_5 (%)	Y_6 (%)	Y_7[c] (%)
1	1.0	5.0	10.0/5.0	DCM	Δ	**50**	**49**	**17**
2*[a]*	1.0	5.0	10.0/5.0	DCM	Δ	**50**	**49**	**17**
3	1.0	5.0	10.0/5.0	DCM	RT	**traces**	-	-
4	1.0	5.0	10.0/5.0	DCE	Δ	**16**	-	-
5	1.0	5.0	10.0/5.0	THF	Δ	-	-	-
6	2.0	1.0	2.2/0.3	DCM	Δ	**12**	-	-

[a] Reactions were carried out overnight under N_2 atmosphere.
[a] Reaction time: 48 h.
[b] Concentration: 0.01 M.
[c] Yield based on **4**.

1.6. Metal scope for 5·EtOAc

Table S3. Coordination conditions tested for **5**·EtOAc with different metals.

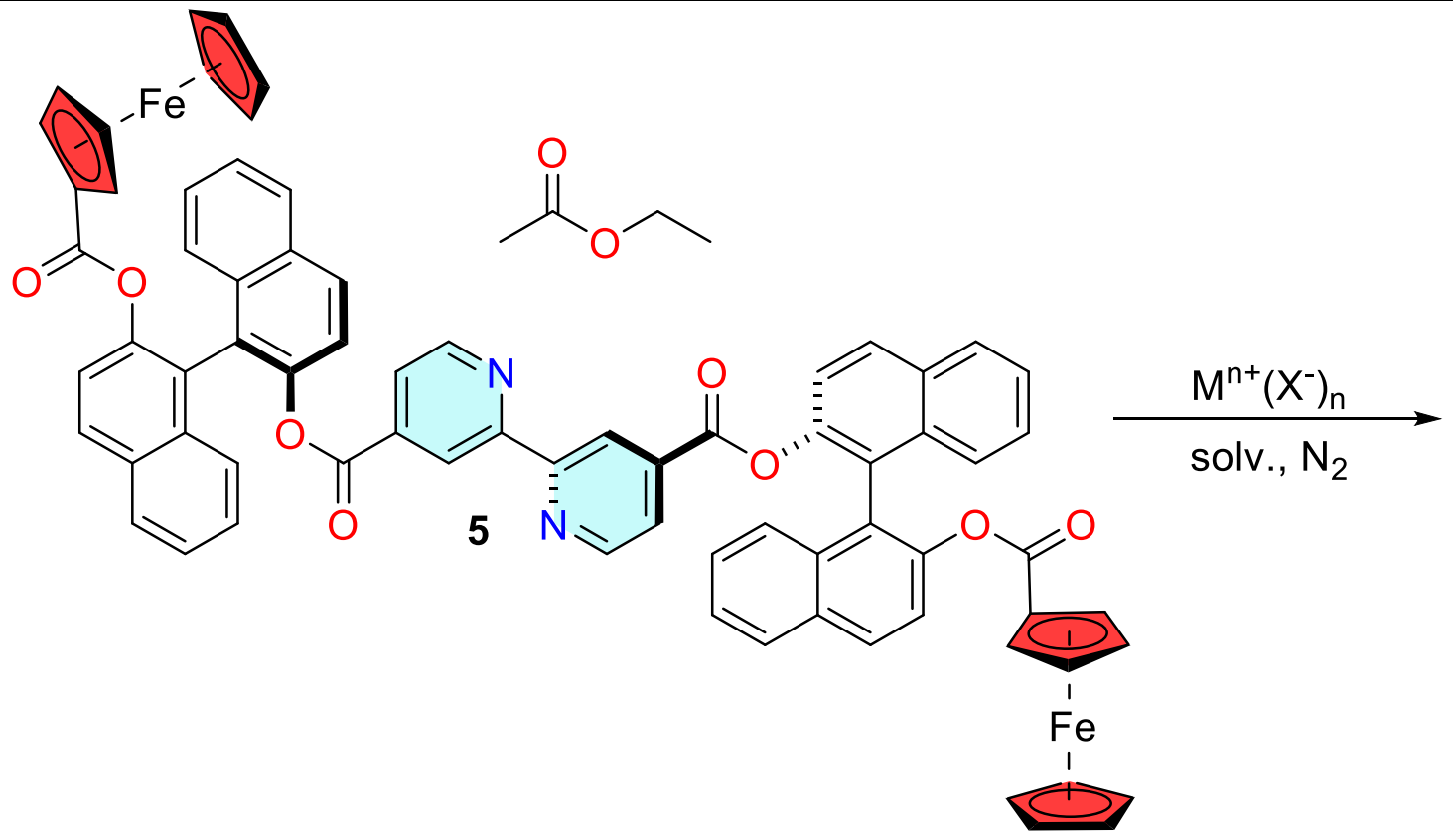

Entry[a]	$M^{n+}(X^-)_n$ (1.0 equiv.)	Solvent (0.05 M)	Colour	Y (%)
1	$CuCl_2$	EtOH	Yellow-orange	-[b]
2	$CuBr_2$	EtOH	Orange	-[b]
3[2]	$FeCl_3$	DCM	Brown	-[b]
4	$Pd(OAc)_2$	ACN	Orange	**NC**
5	$PdCl_2(ACN)_2$	ACN	Orange	**NC**
6	$Er(OTf)_3$	ACN	Red-orange	-[b]
7	$Yb(OTf)_3$	ACN	Red-orange	-[b]

[2] Conditions inspired by: Amani, V.; Safari, N.; Khavasi, H. R. Synthesis, Characterization and Crystal Structure Determination of Iron(III) Hetero-Ligand Complexes Containing 2,2′-Bipyridine, 5,5′-Dimethyl-2,2′-Bipyridine and Chloride, $[Fe(Bipy)Cl_4][Bipy{\cdot}H]$ and $[Fe(Dmbipy)_2Cl_2][FeCl_4]$. *Polyhedron* **2007**, *26* (15), 4257–4262.

[a] Reactions were carried out at room temperature under an N_2 atmosphere overnight.
[b] The crystals that formed corresponded to **3**. NC = No crystals obtained.

1.7. Metal scope for 6

Table S4. Coordination conditions tested for **6** with different metals.

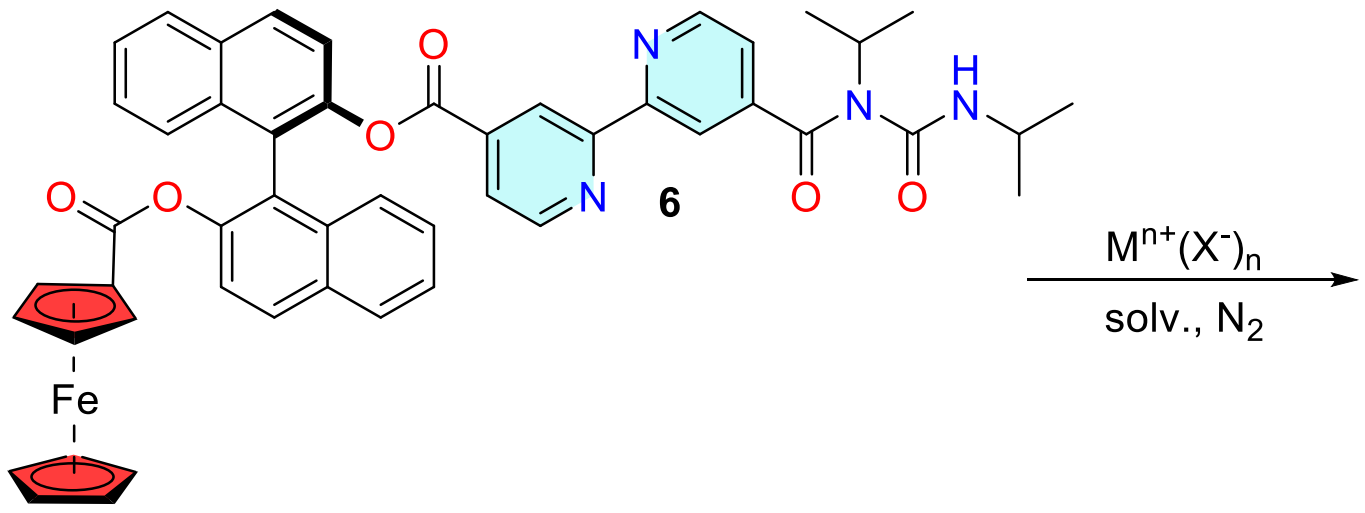

Entry[a]	$M^{n+}(X^-)_n$ (1.0 equiv.)	Solvent (0.05 M)	Colour	Y (%)
1	$CuCl_2$	EtOH	Yellow-orange	-[b]
2	$CuBr_2$	EtOH	Orange	-[b]
3[2]	$FeCl_3$	DCM	Brown	-[b]
4	$Pd(OAc)_2$	ACN	Orange	**NC**
5	$PdCl_2(ACN)_2$	ACN	Orange	**NC**
6	$Tb(OTf)_3$	ACN	Red-orange	-[b]
7	$Yb(OTf)_3$	ACN	Red-orange	-[b]

[a] Reactions were carried out at room temperature under an N_2 atmosphere overnight.
[b] The crystals that formed corresponded to **3**. NC = No crystals obtained.

1.8. Metal scope for 7

Table S5. Coordination conditions tested for **7** with different metals.

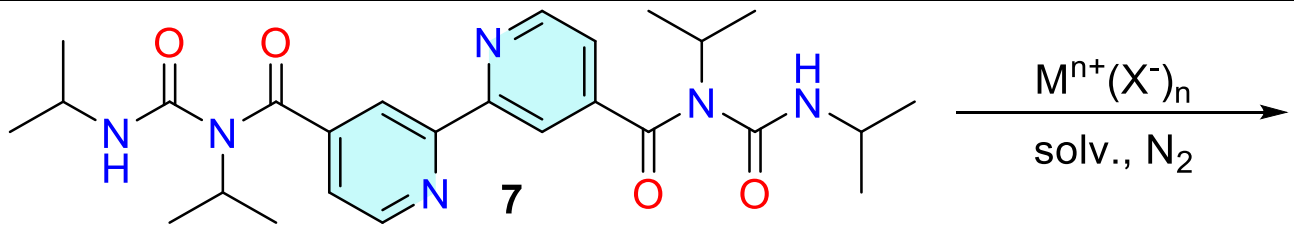

Entry[a]	$M^{n+}(X^-)_n$ (1.0 equiv.)	Solvent (0.05 M)	Colour	Y[b] (%)	CCDC
1	CuCl	EtOH	Pale-yellow	**NC**	-
2	CuBr	EtOH	Orange	**NC**	-
3	CuI	EtOH	Orange	**NC**	-
4	$CuCl_2$	EtOH	Green	**>99**	**2535803**
5	$CuBr_2$	EtOH	Brown	**NC**	-

[a] Reactions were carried out at room temperature under an N_2 atmosphere overnight.
[b] NC = No crystals obtained.

2. NMR spectra

Ferrocene, [[[(S)-(2'-hydroxy-[1,1'-binaphthalen]-2-yl]oxy]carbonyl]- (**3**)

See procedure

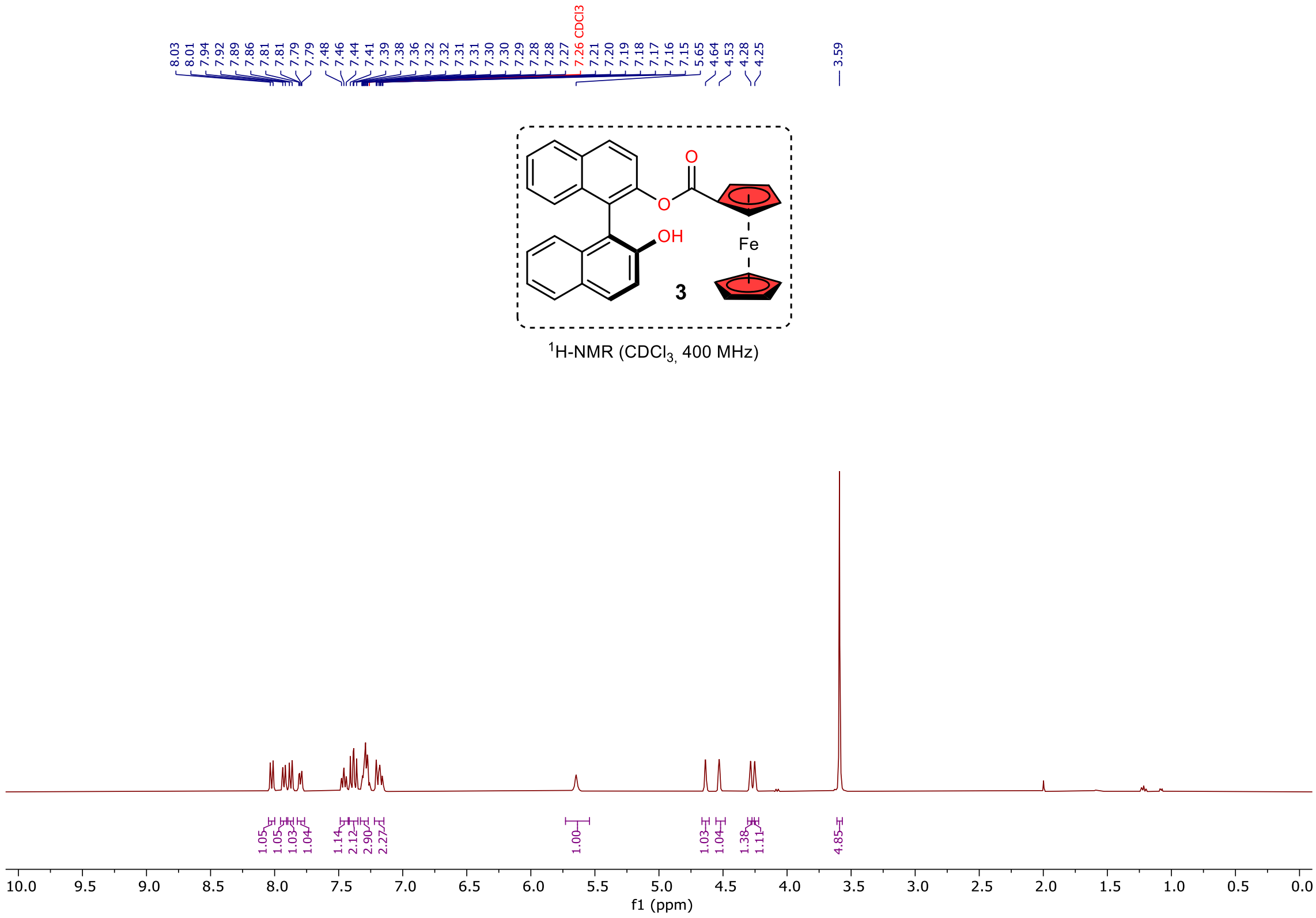

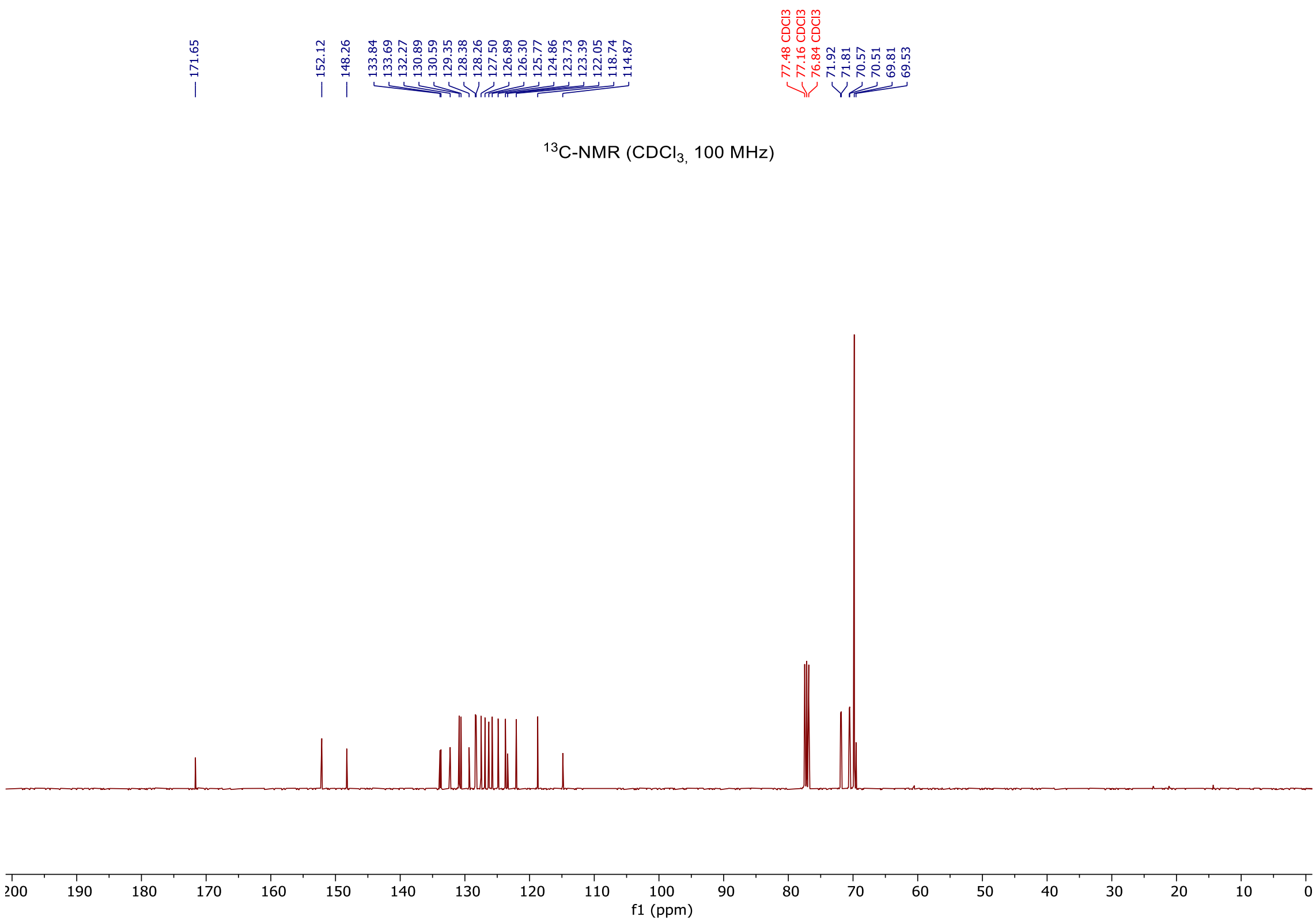

Ferrocene, [[(1-methylethyl)[[(1-methylethyl)amino]carbonyl]amino]carbonyl]- (**3b**)

See procedure

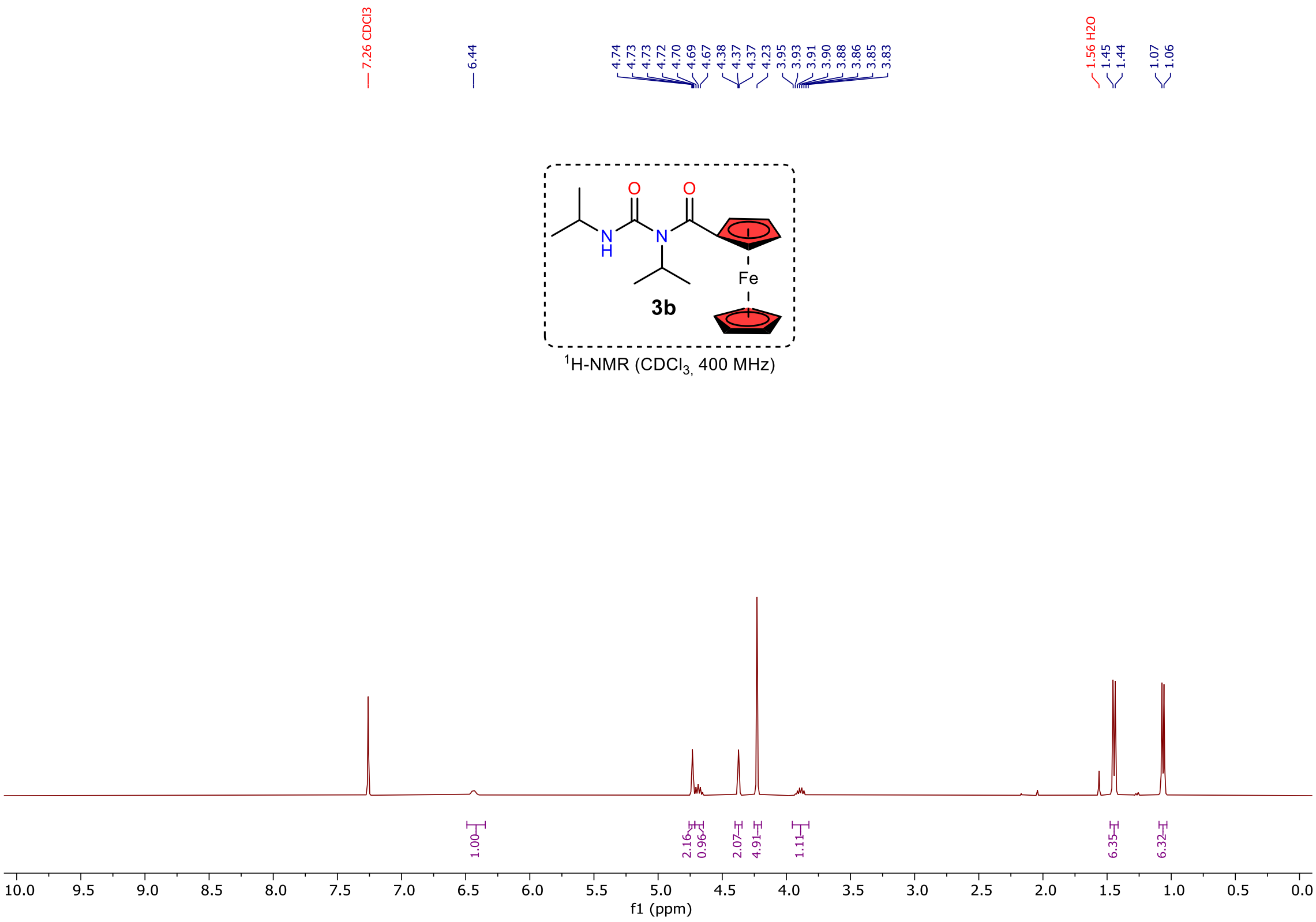

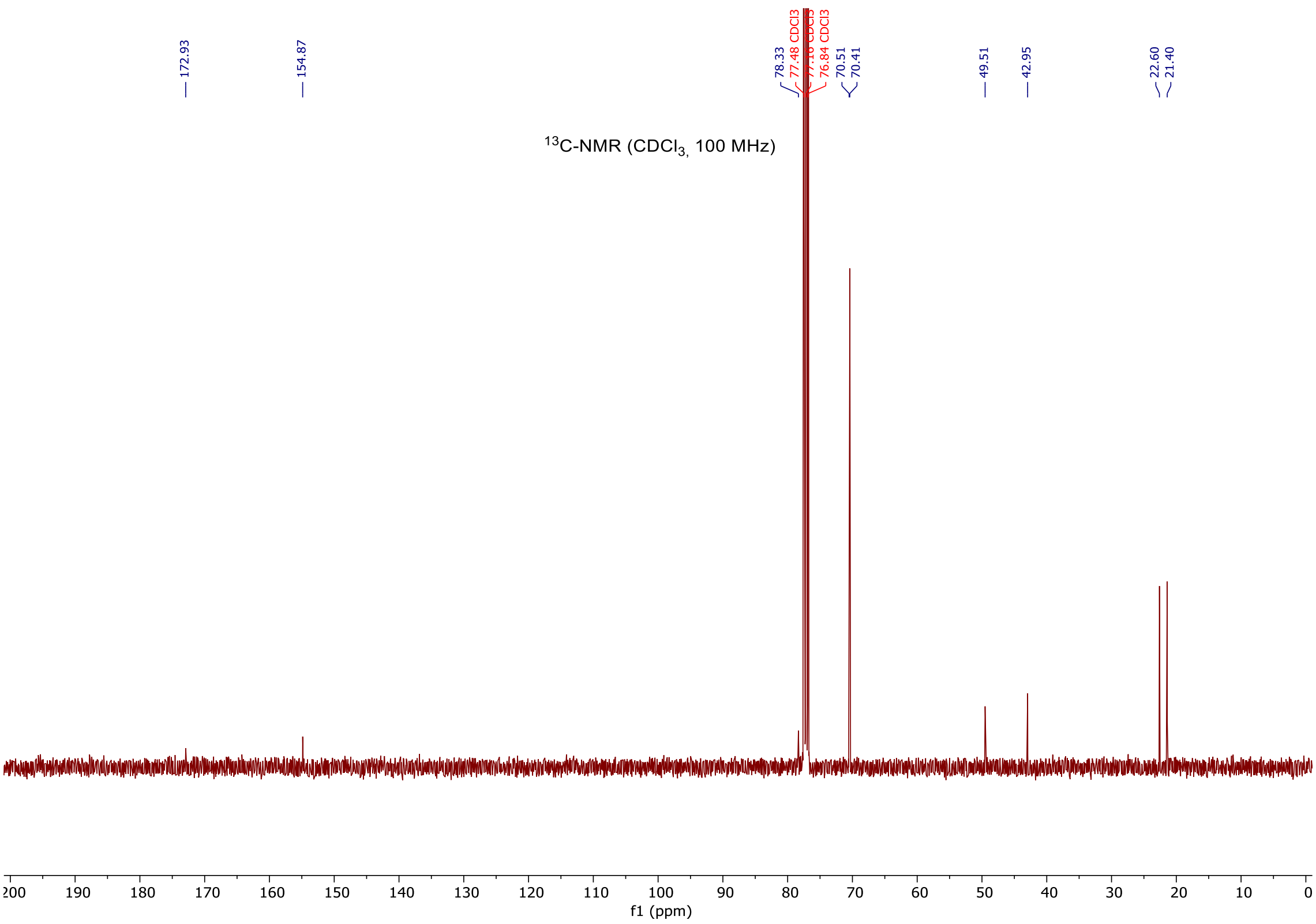

bis((*S*)-2'-((ferrocenecarbonyl)oxy)-[1,1'-binaphthalen]-2-yl) [2,2'-bipyridine]-4,4'-dicarboxylate (**5**·EtOAc)

See procedure

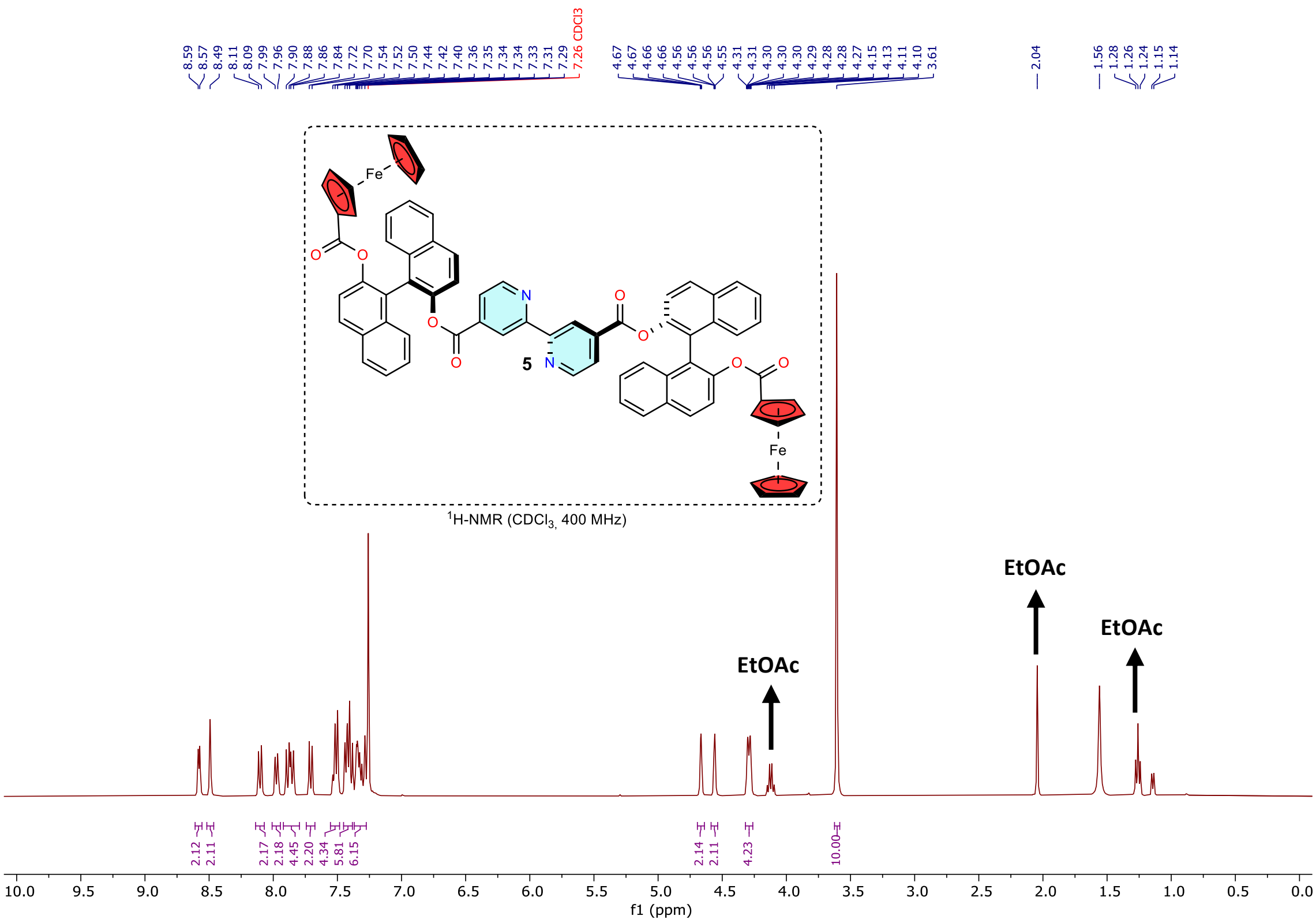

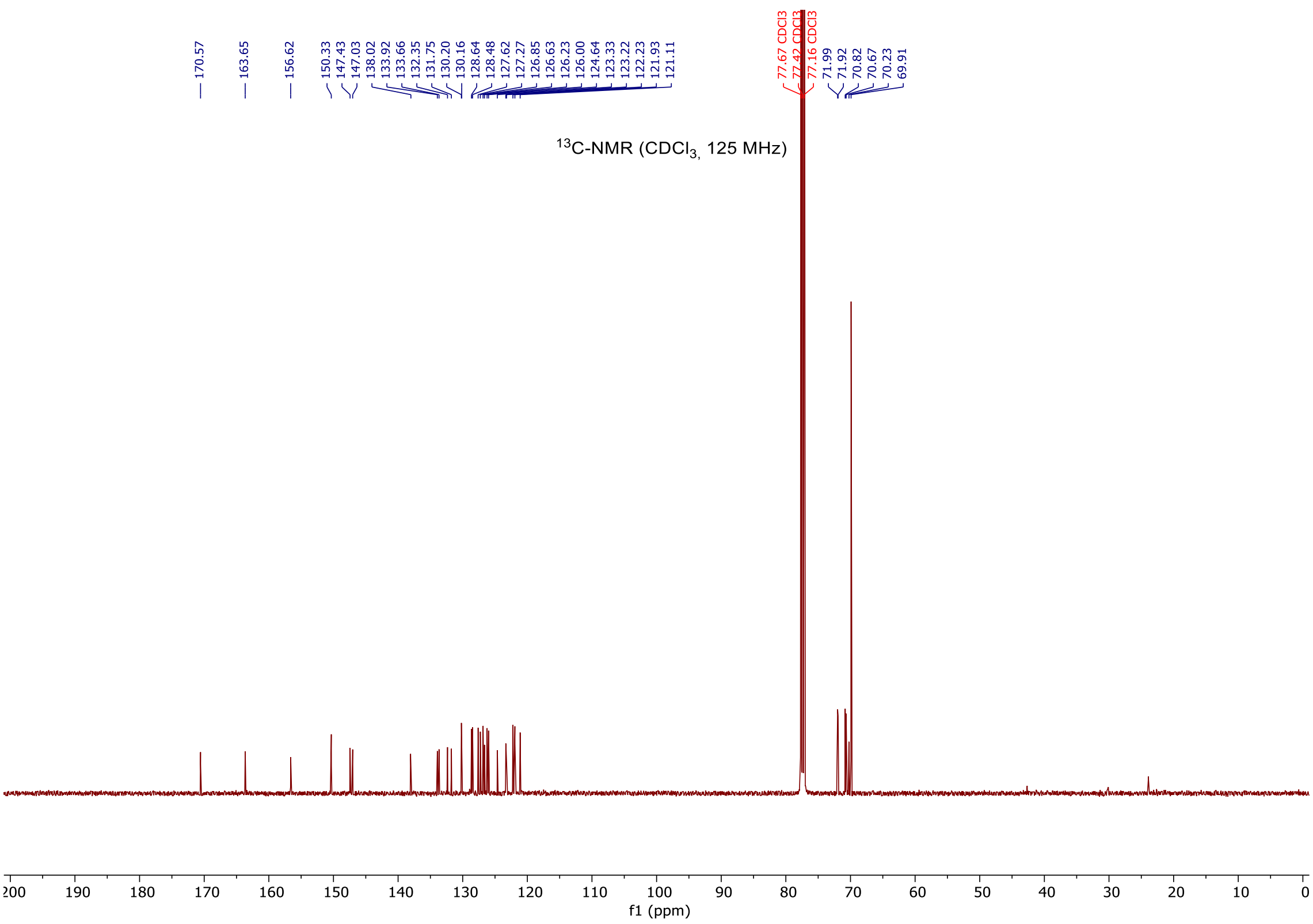

(*S*)-2'-((ferrocenecarbonyl)oxy)-[1,1'-binaphthalen]-2-yl 4'-(isopropyl(isopropylcarbamoyl)carbamoyl)-[2,2'-bipyridine]-4-carboxylate (**6**)

See procedure

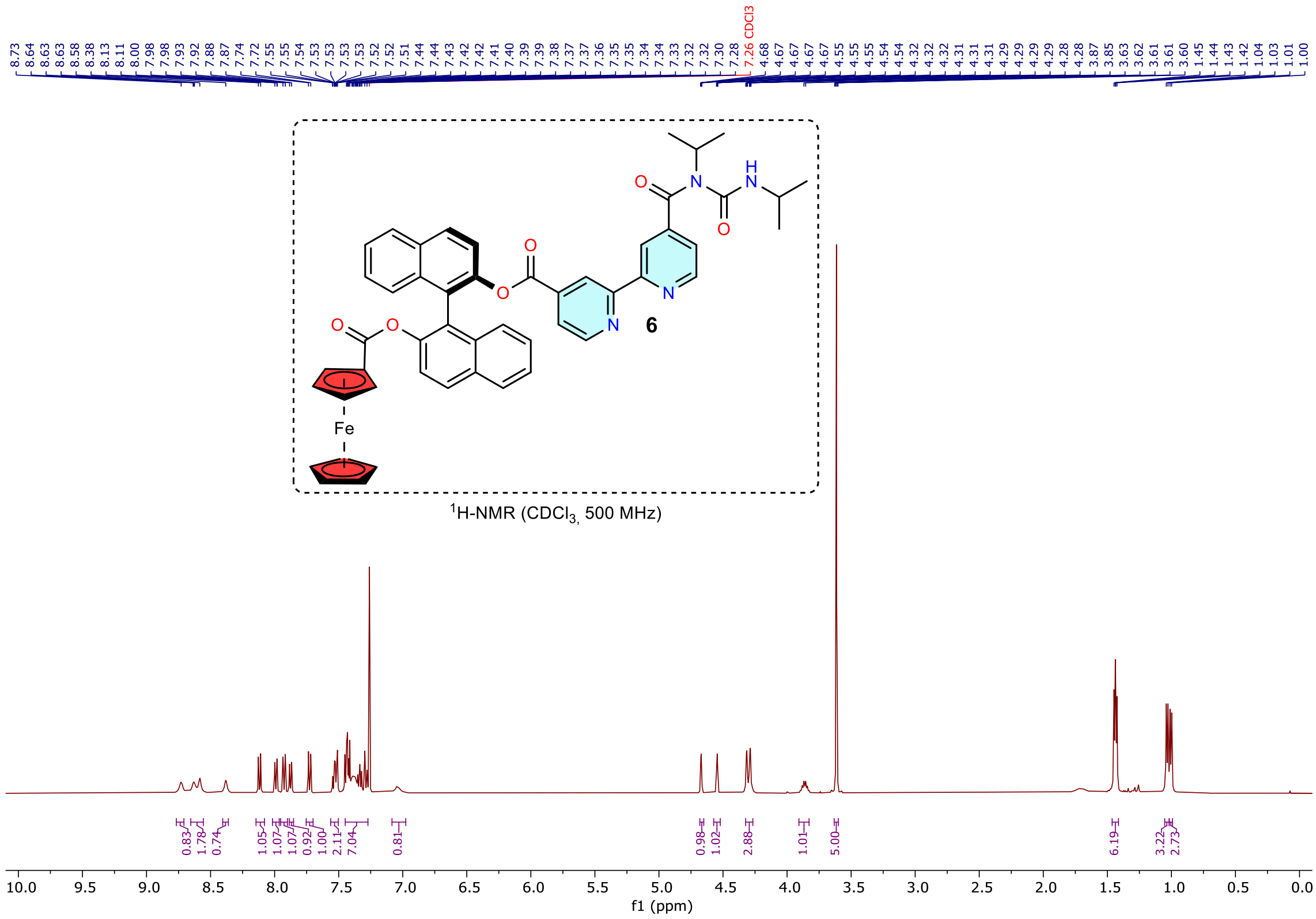

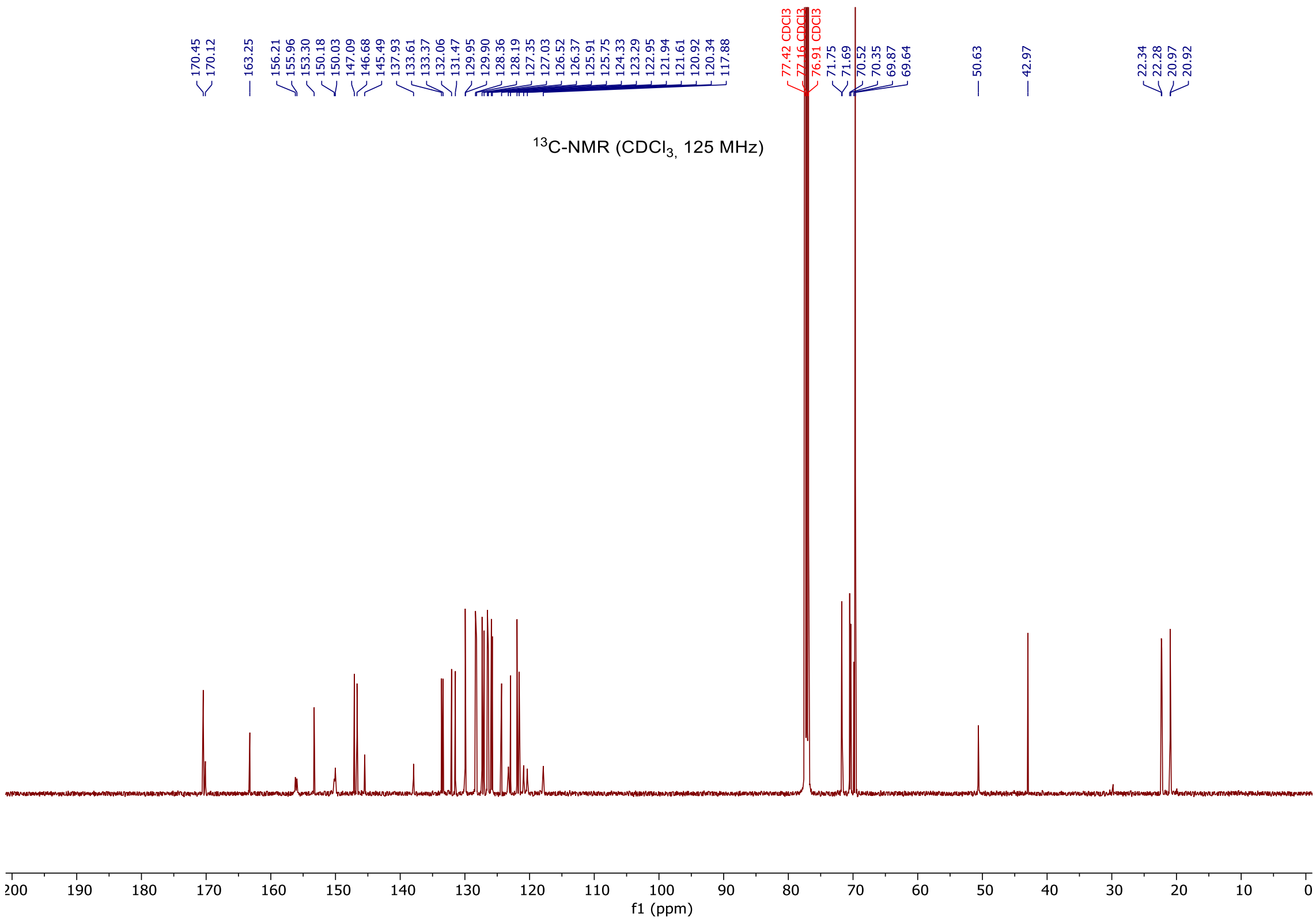

3. Infrared analysis of 8·$(H_2O)_4$

Transmittance measurements were conducted using an ATR PRO ONE spectrometer equipped with a TGS detector, employing an accumulation of 64 scans and a resolution of 8 cm^{-1}. The aperture size was set to 7.1 mm, with a scanning speed of 2 mm/sec and a filter frequency of 10000 Hz.

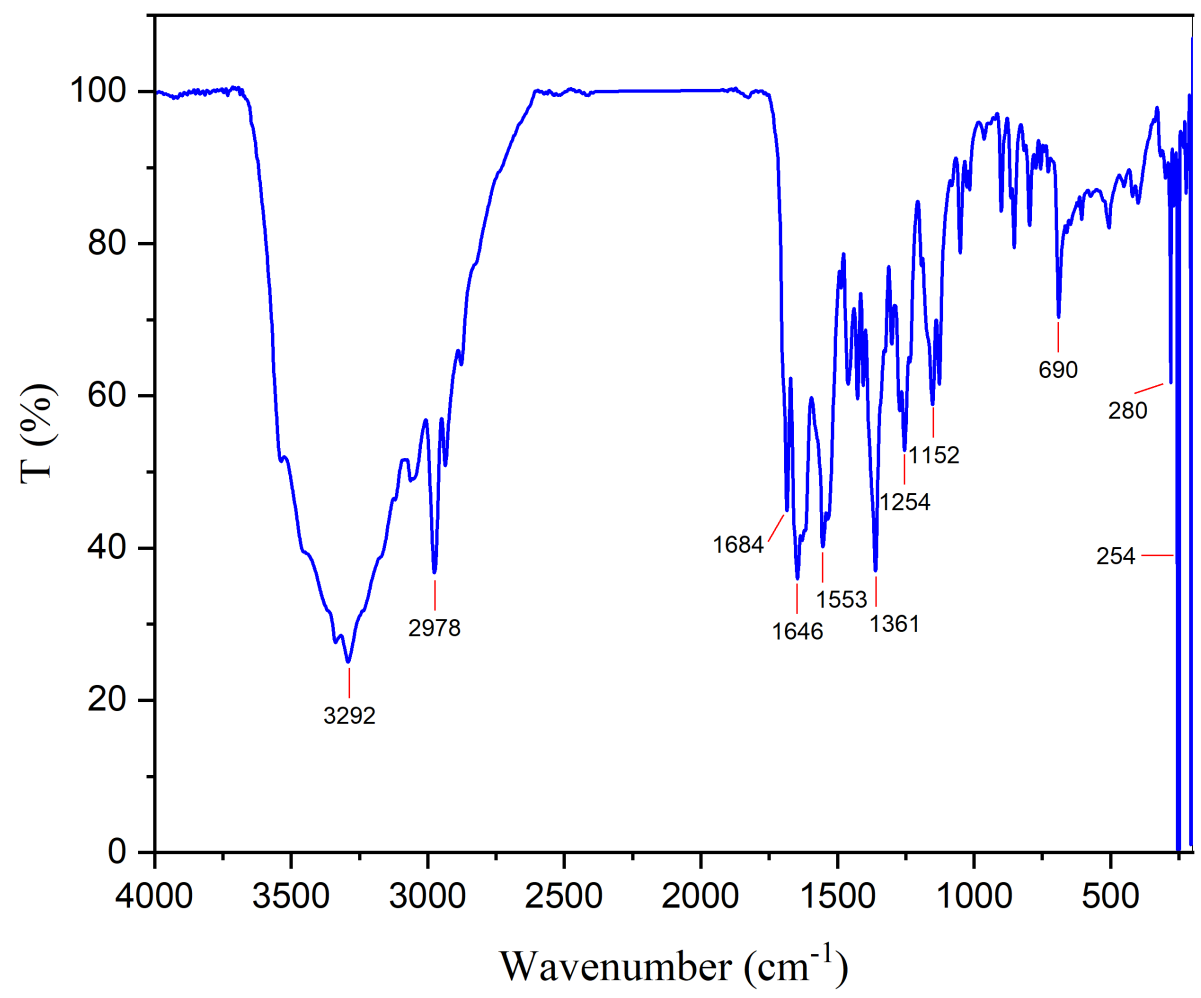

Figure S1. IR spectrum of transmittance (%) for **8**·$(H_2O)_4$**.**

Table S6. A selection of FT-IR (cm^{-1}) data of characteristic vibrations.[3]

FT-IR band (cm^{-1})	Vibrational mode
3292	$\upsilon(H_2O)$, υ(NH)
2978	$\upsilon(C\text{-}H)_{aliphatic}$
1684, 1646	υ(C=O)
1553	υ(C-H)
1361	δ(C-N-H)
1254	υ(C-N)
1152	$\upsilon(C\text{-}H)_{aromatic}$
690	υ(Cu-N)
280	$\upsilon(Cu\text{-}Cl)_{terminal}$
254	υ(Cu-μ-Cl)

[3] (1) Yang, Q.; Cai, J.; Li, G.; Gao, R.; Han, Z.; Han, J.; Liu, D.; Song, L.; Shi, Z.; Wang, D.; Wang, G.; Zheng, W.; Zhou, G.; Song, Y. *Nat. Commun.* **2024**, *15* (1), 1–13. (2) Davydov, V. V.; Sokol, V. I.; Kolyadina, N. M.; Polyakova, E. I.; Chernov, S. V.; Shklyaev, Y. V.; Sergienko, V. S. *Russ. J. Inorg. Chem.* **2008**, *53* (10), 1573–1580. (3) Wang, J.; Djukic, B.; Cao, J.; Alberola, A.; Razavi, F. S.; Pilkington, M. *Inorg. Chem.* **2007**, *46* (21), 8560–8568. (4) Singh, B. K.; Bhojak, N.; Mishra, P.; Garg, B. S. *Spectrochim. Acta - Part A Mol. Biomol. Spectrosc.* **2008**, *70* (4), 758–765.

4. Thermal Analysis

Thermograms of TGA and DSC were obtained using a simultaneous TGA/DSC Discovery SDT 650 (TA Instruments). Measurements were performed with hermetically crimped Tzero aluminum crucibles (40 µL, 6.554427 mg of **5**·EtOAc, 5.207981 mg of **6** and 4.300264 mg of **8·$(H_2O)_4$** under nitrogen atmosphere at a flow rate of 50 mL/min^{-1} from 25 to 500 °C (at a heating rate of 10 C min^{-1}). The number of decomposition steps was identified using the derivative thermogravimetric curve (DTG).

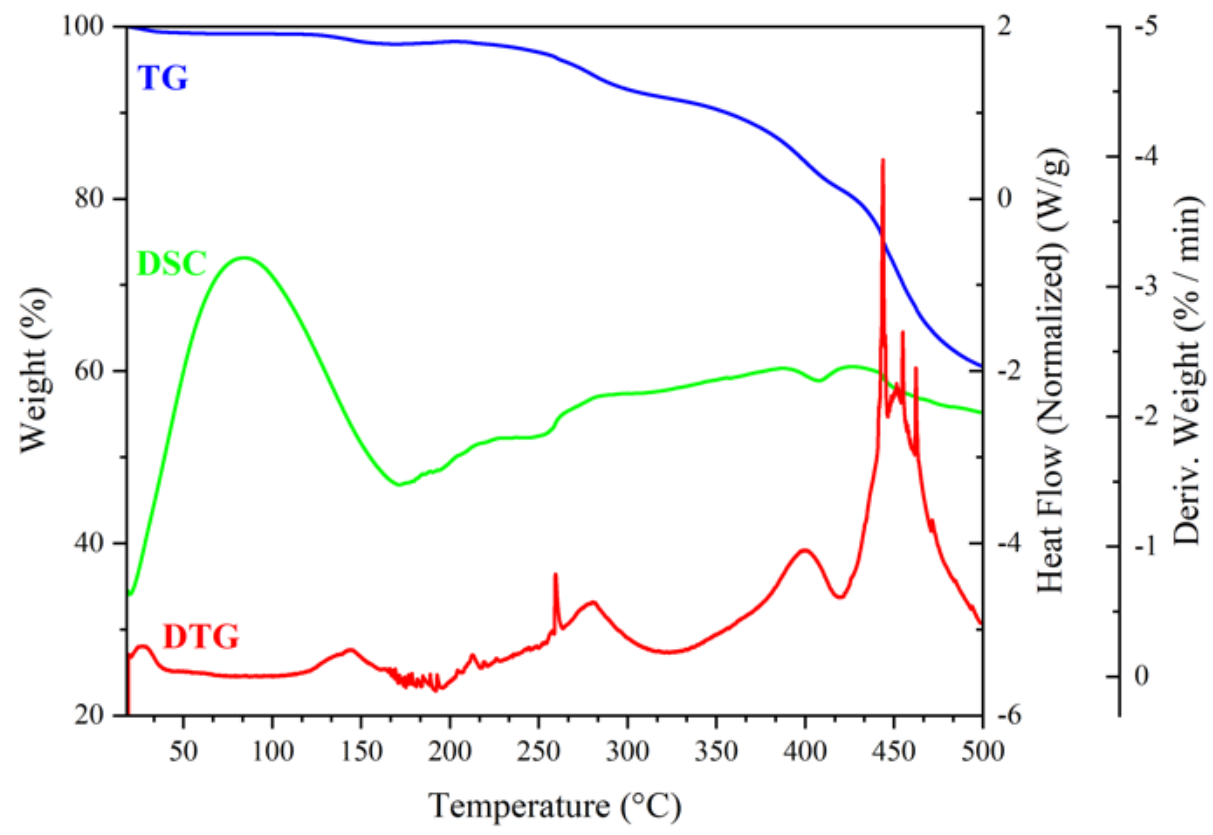

Figure S2. TG (blue), DSC (green) and DTG (red) curves of **5**·EtOAc.

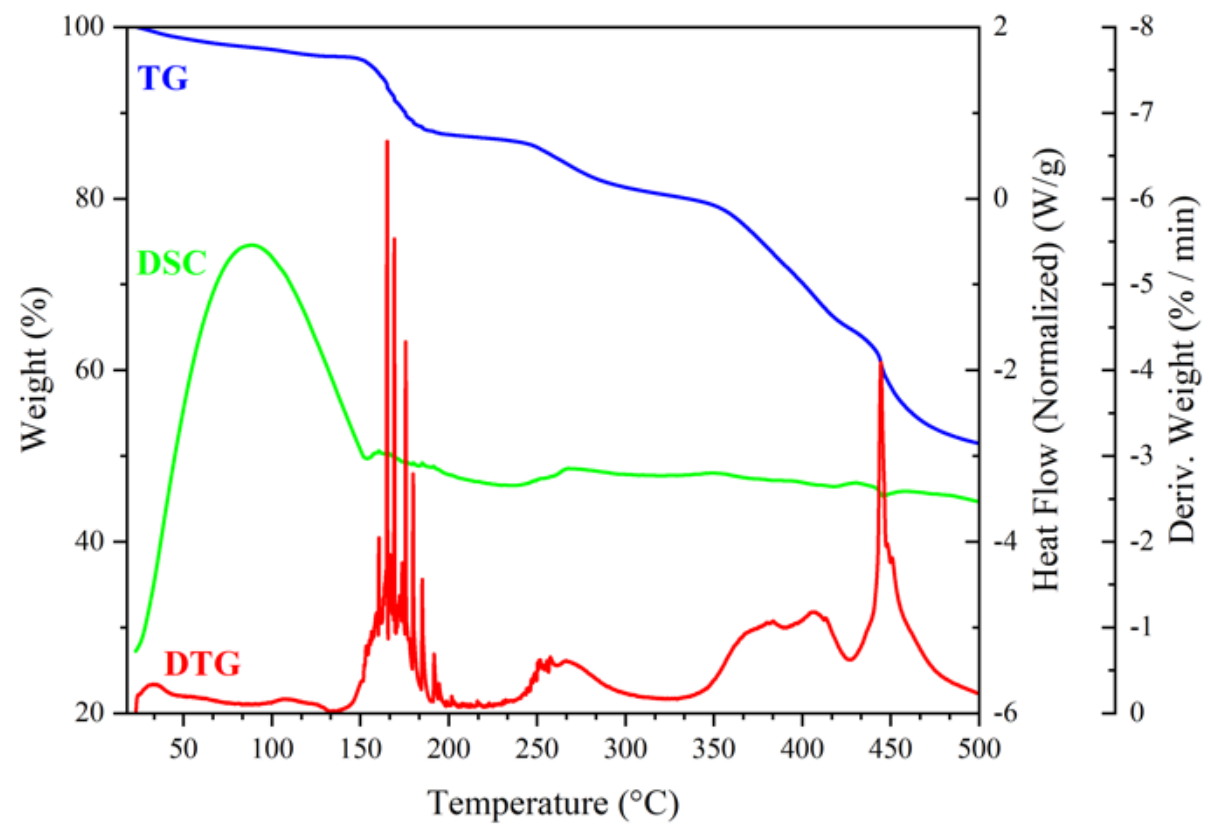

Figure S3. TG (blue), DSC (green) and DTG (red) curves of **6**.

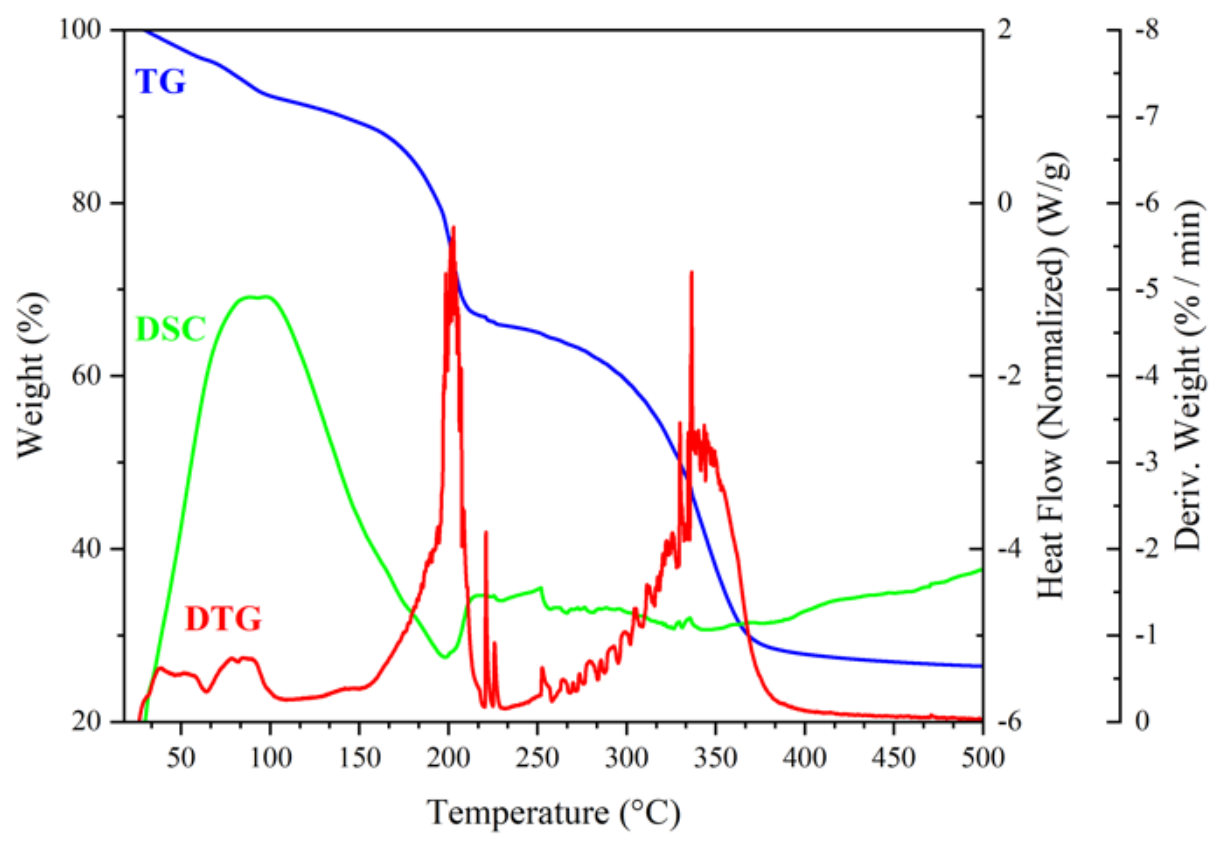

Figure S4. TG (blue), DSC (green) and DTG (red) curves of **8·$(H_2O)_4$**.

5. ESI-MS measurements of 8·$(H_2O)_4$

A solution of 2.7 mg of **8·$(H_2O)_4$**; L = 7 in 10 mL of MS-grade methanol was prepared and injected into a Waters SYNAPT XS ion mobility Q-TOF mass spectrometer operating in positive mode. The fragment **$[Cu_2Cl_3(7)_2]^+$** was detected, corresponding to a chloride-loss adduct of the complex.

Table S7. Adduct detected by ESI(+)-QTOF MS of **8·$(H_2O)_4$**.

Adduct	Formula	*m/z* calc.	*m/z* exp
**$[Cu_2Cl_3(7)_2]^+$**	$C_{52}H_{72}N_{12}O_8{}^{35}Cl_3{}^{63}Cu_2$	1223.3248	1223.3239
	$C_{52}H_{72}N_{12}O_8{}^{35}Cl_2{}^{37}Cl^{63}Cu_2$	1225.3219	1225.3225
	$C_{52}H_{72}N_{12}O_8{}^{35}Cl^{37}Cl_2{}^{63}Cu_2$	1227.3189	1227.3196
	$C_{52}H_{72}N_{12}O_8{}^{35}Cl_2{}^{37}Cl^{65}Cu_2$	1229.3183	1229.3193

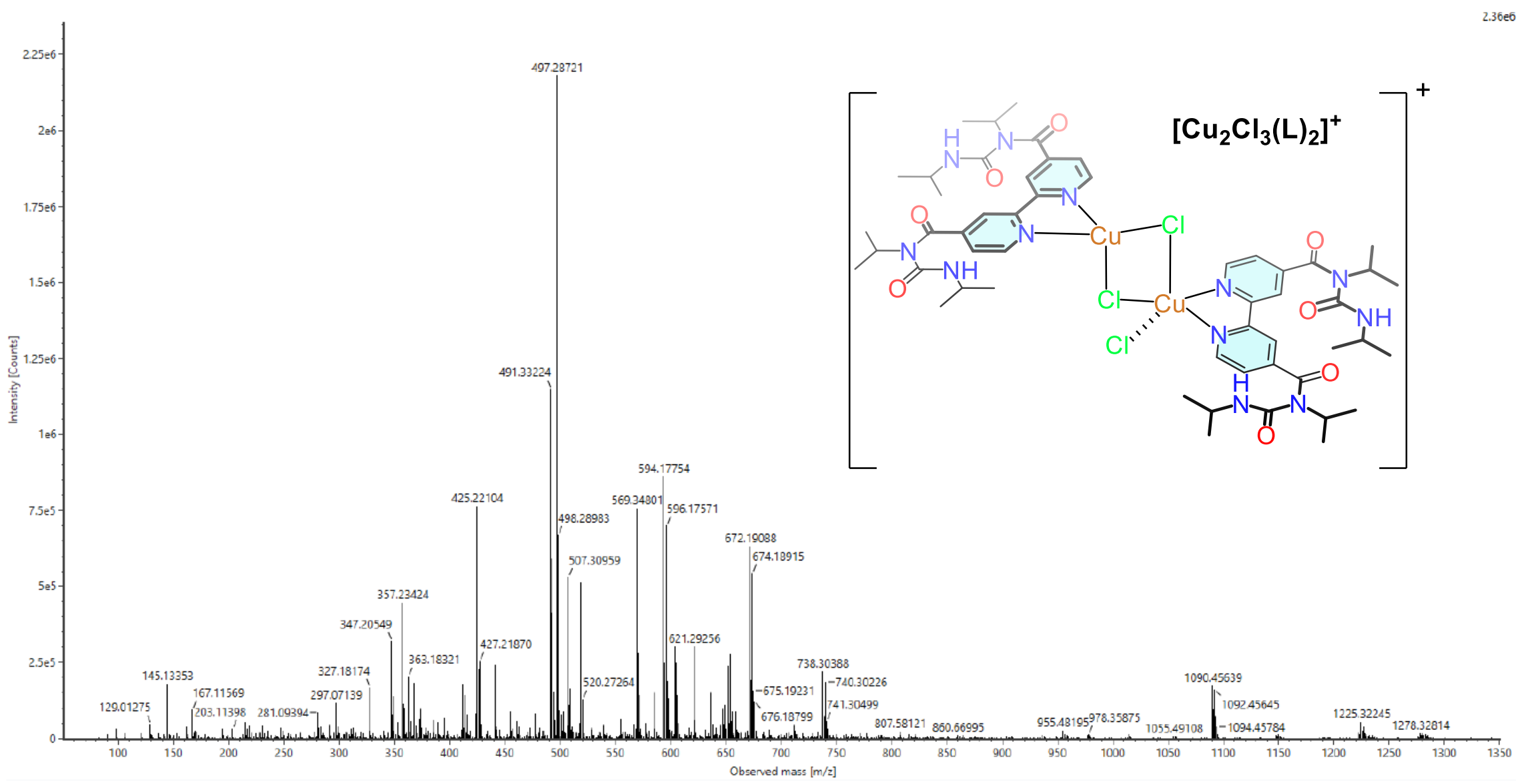

Figure S5. Experimental profile of cationic adducts for **8·$(H_2O)_4$**.

6. Optical characterization

6.1. (*S*)-BINOL (**1**)

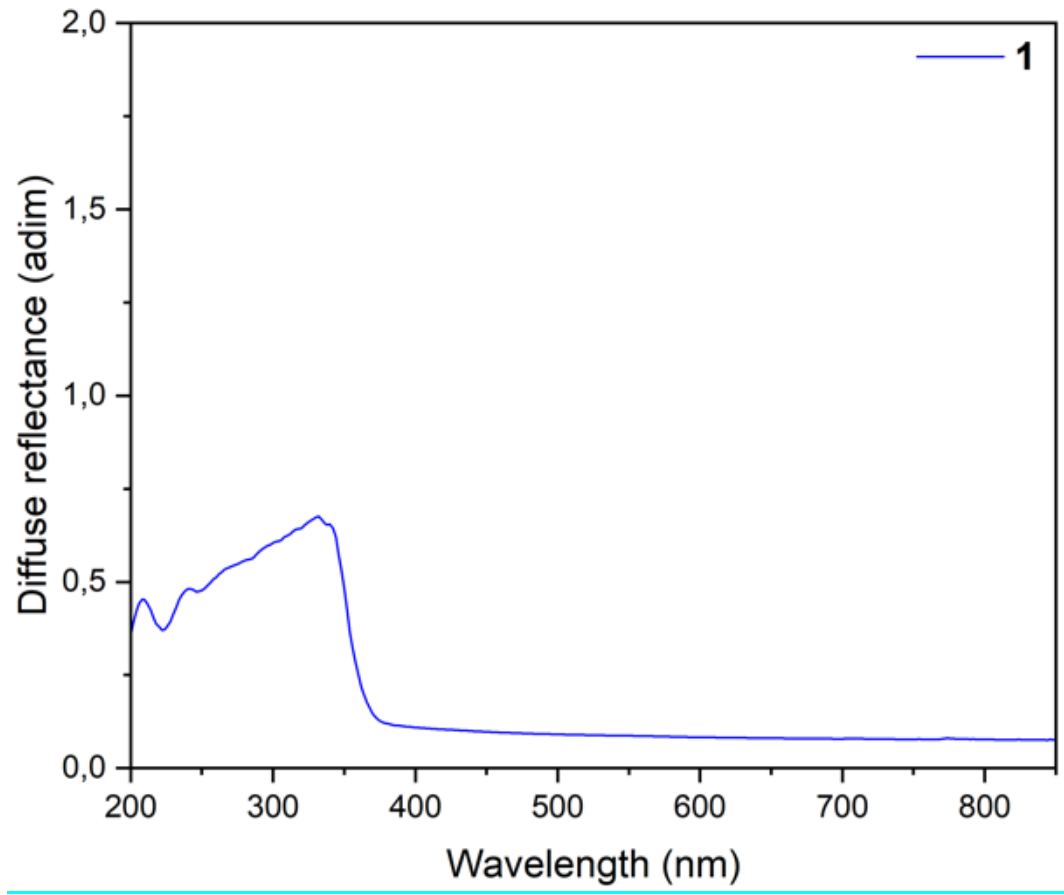

Figure S6. Diffuse reflectance spectra of (*S*)-BINOL (**1**).

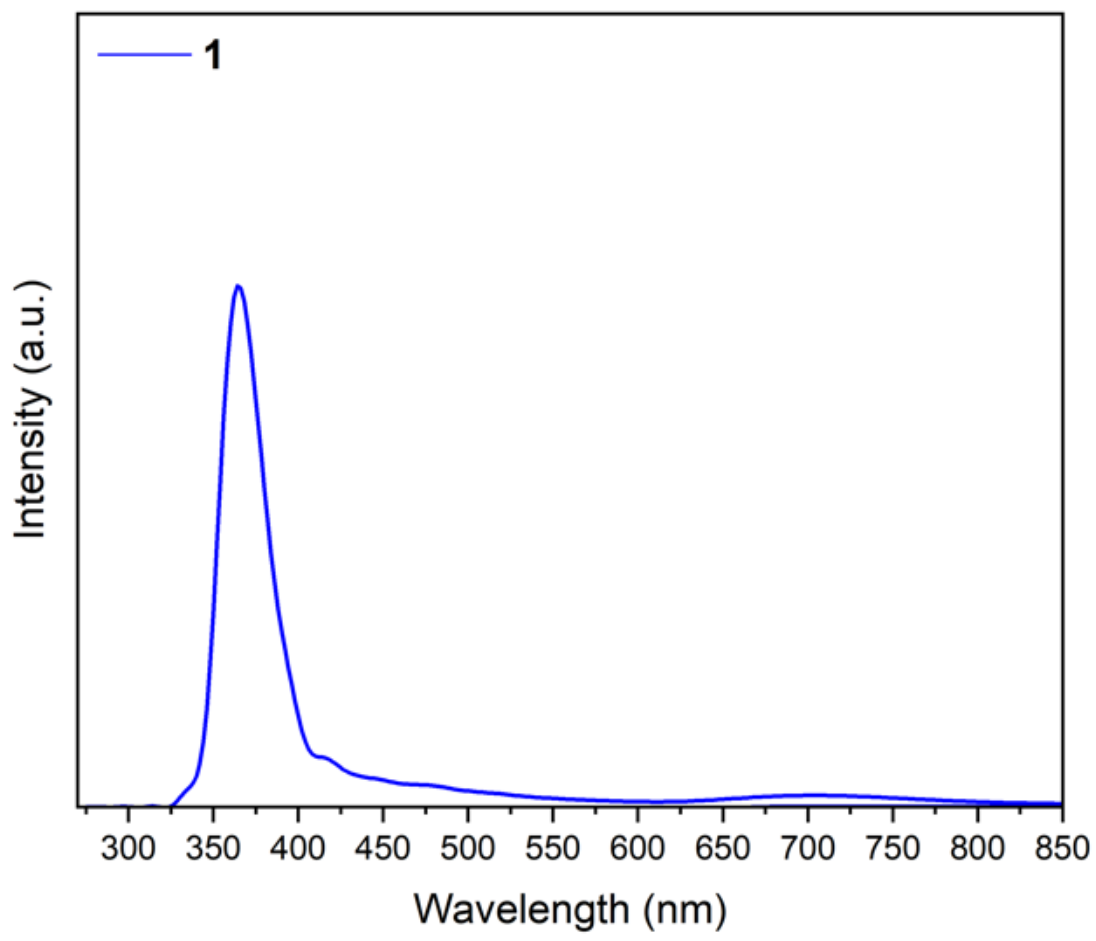

Figure S7. Emission spectra of (*S*)-BINOL (**1**) recorded upon excitation at 250 nm using a xenon lamp and a 395 nm cutoff filter. a.u. = arbitrary units.

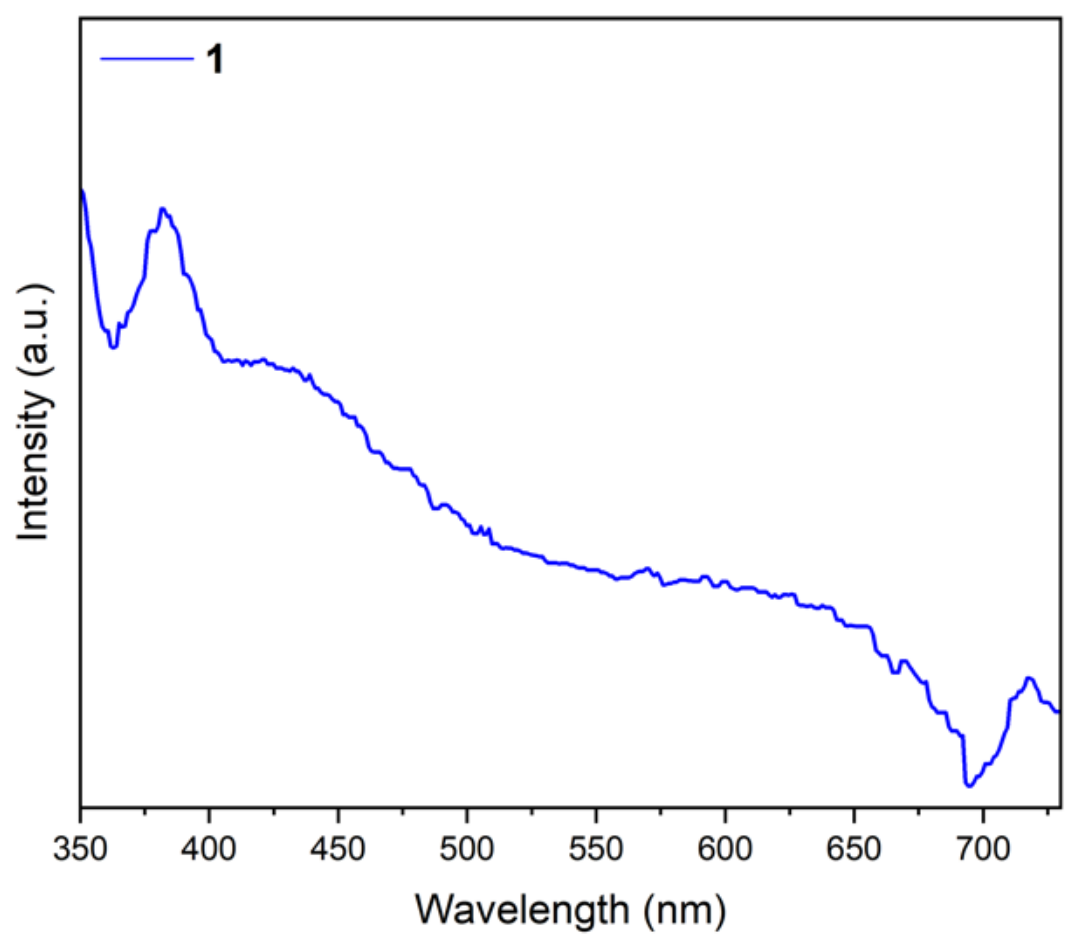

Figure S8. Excitation spectra of (*S*)-BINOL (**1**) monitored at an emission wavelength of 750 nm and recorded without optical filters. a.u. = arbitrary units.

6.2. Ferrocenecarboxylic acid (**2**)

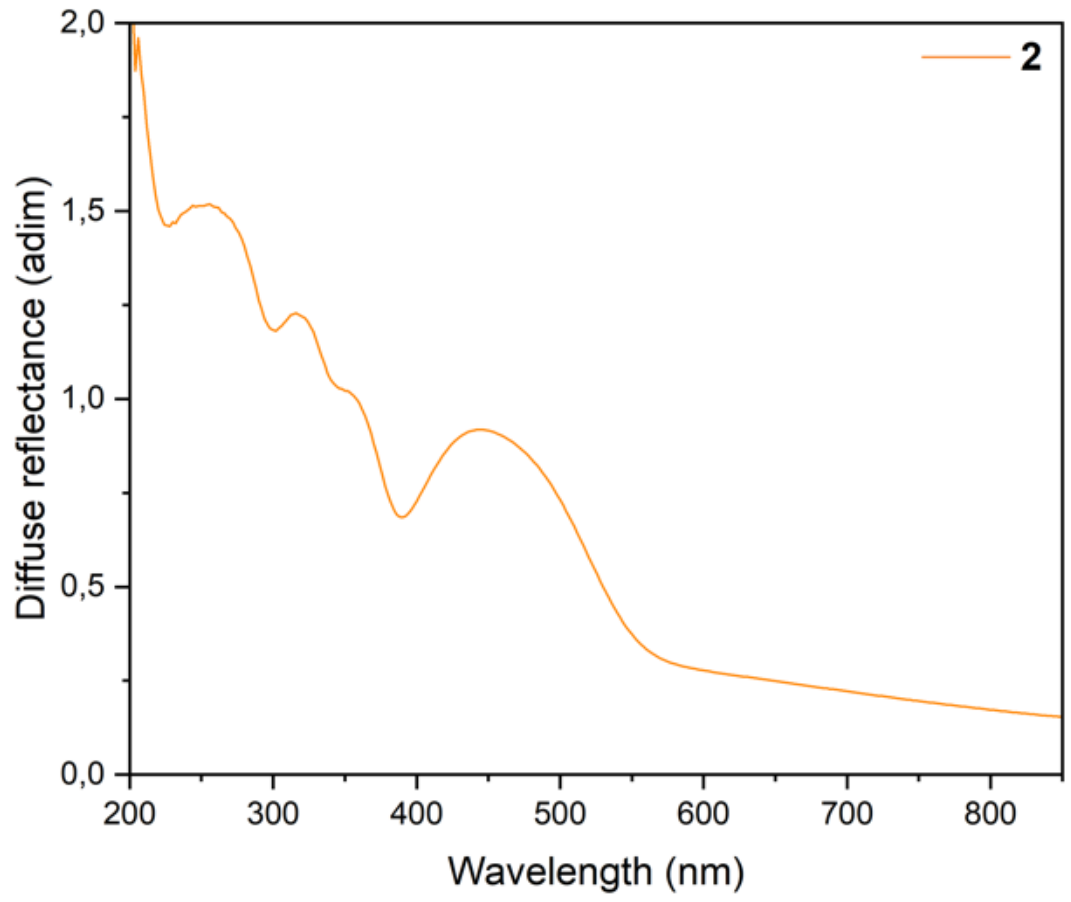

Figure S9. Diffuse reflectance spectra of ferrocenecarboxylic acid (**2**).

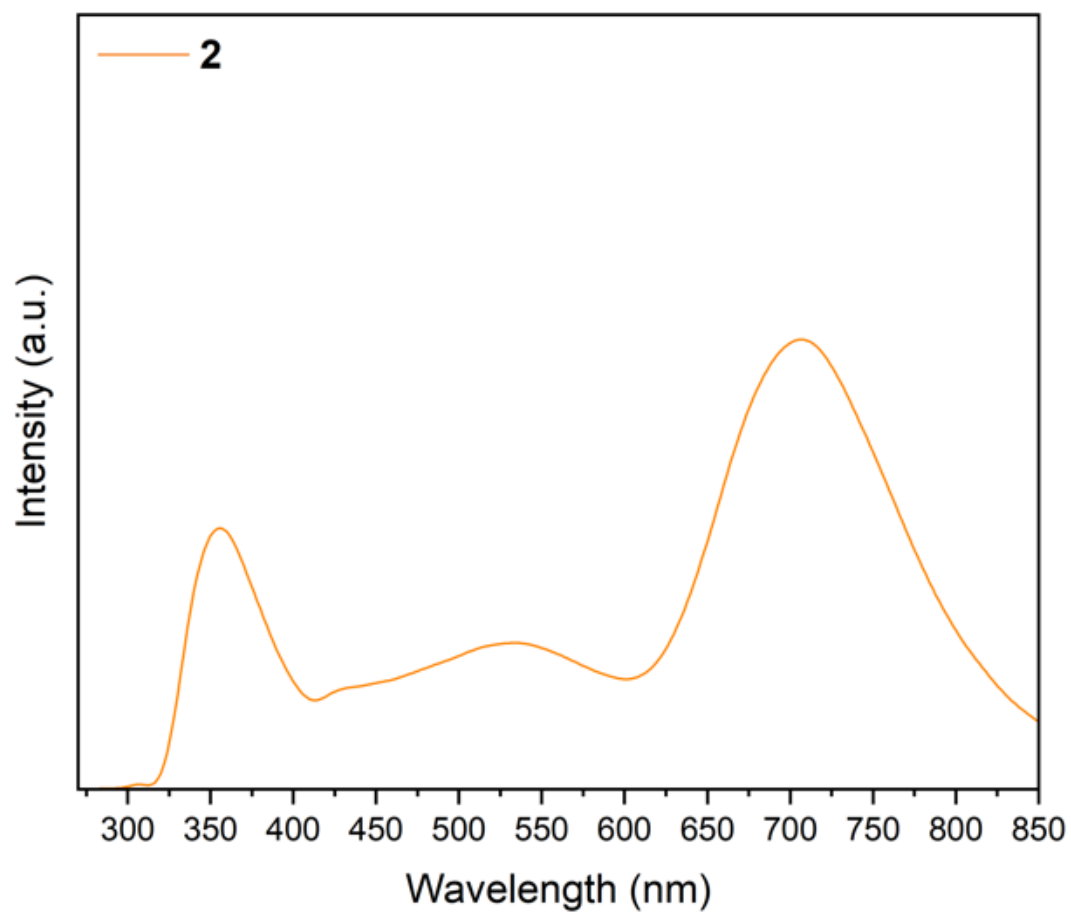

Figure S10. Emission spectra of ferrocenecarboxylic acid (**2**) recorded upon excitation at 250 nm using a xenon lamp and a 395 nm cutoff filter. a.u. = arbitrary units.

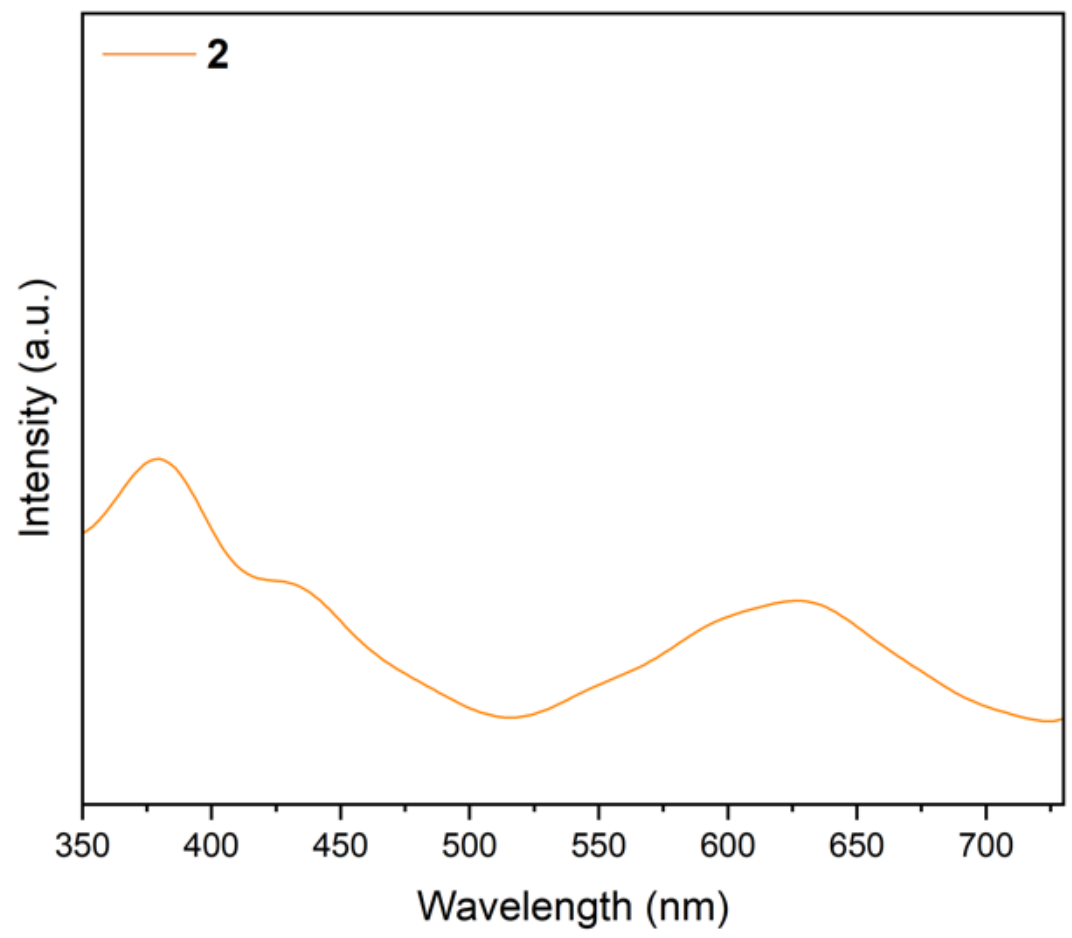

Figure S11. Excitation spectra of ferrocenecarboxylic acid (**2**) monitored at an emission wavelength of 750 nm and recorded without optical filters. a.u. = arbitrary units.

6.3. Ferrocene, [[[(*S*)-(2'-hydroxy-[1,1'-binaphthalen]-2-yl]oxy]carbonyl]- (**3**)

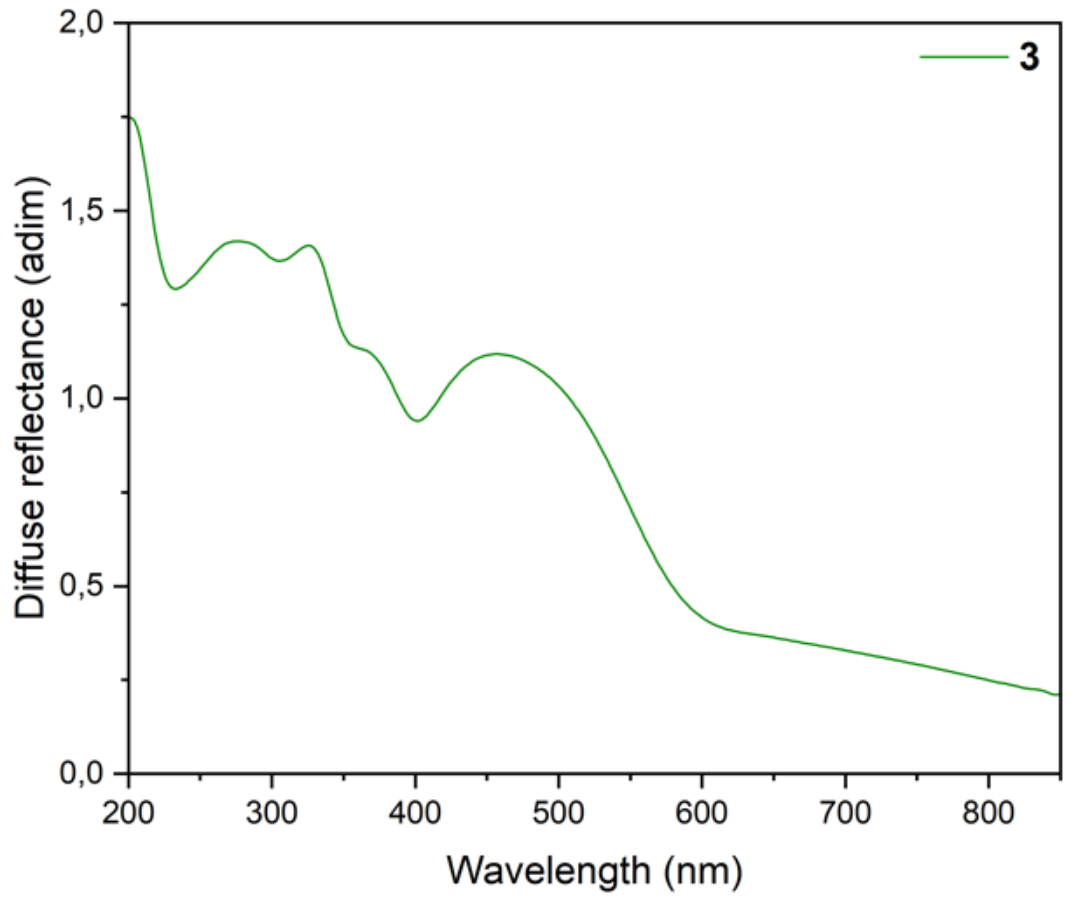

Figure S12. Diffuse reflectance spectra of ferrocene, [[[(*S*)-(2'-hydroxy-[1,1'-binaphthalen]-2-yl]oxy]carbonyl]- (**3**).

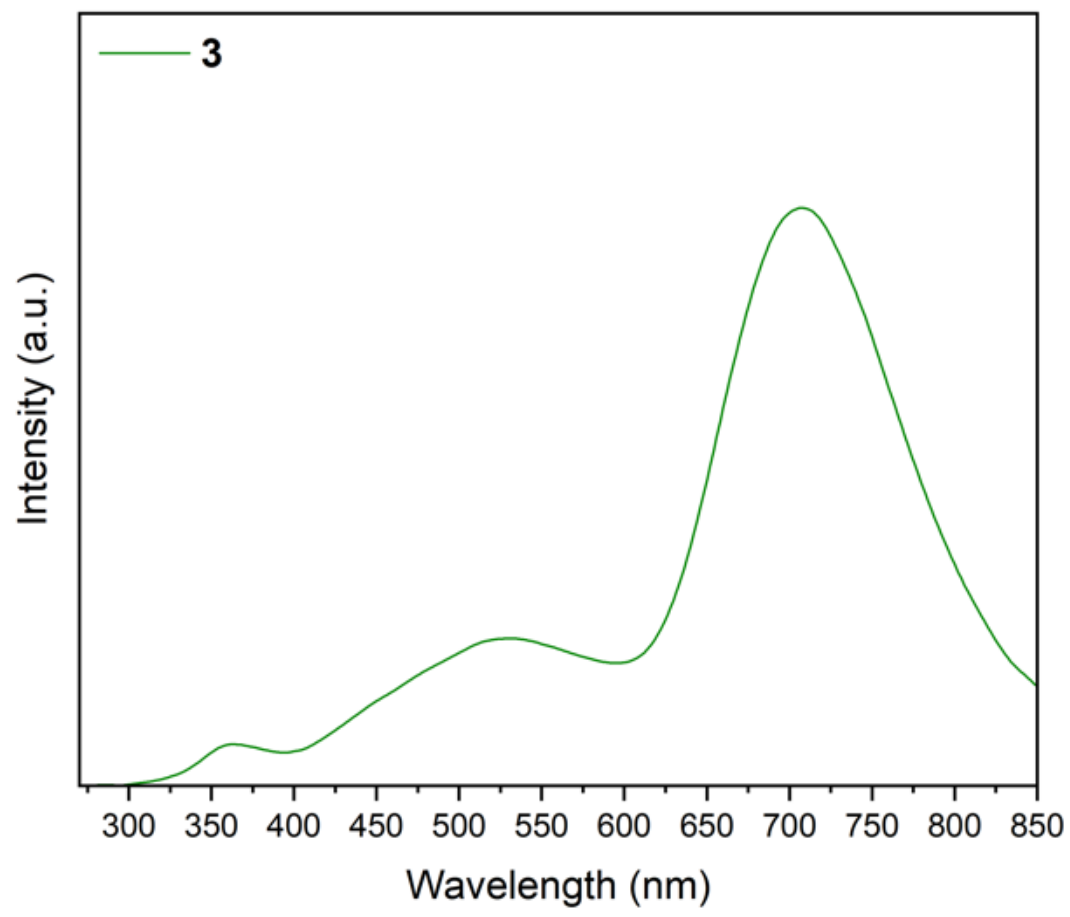

Figure S13. Emission spectra of ferrocene, [[[(*S*)-(2'-hydroxy-[1,1'-binaphthalen]-2-yl]oxy]carbonyl]- (**3**) recorded upon excitation at 250 nm using a xenon lamp and a 395 nm cutoff filter. a.u. = arbitrary units.

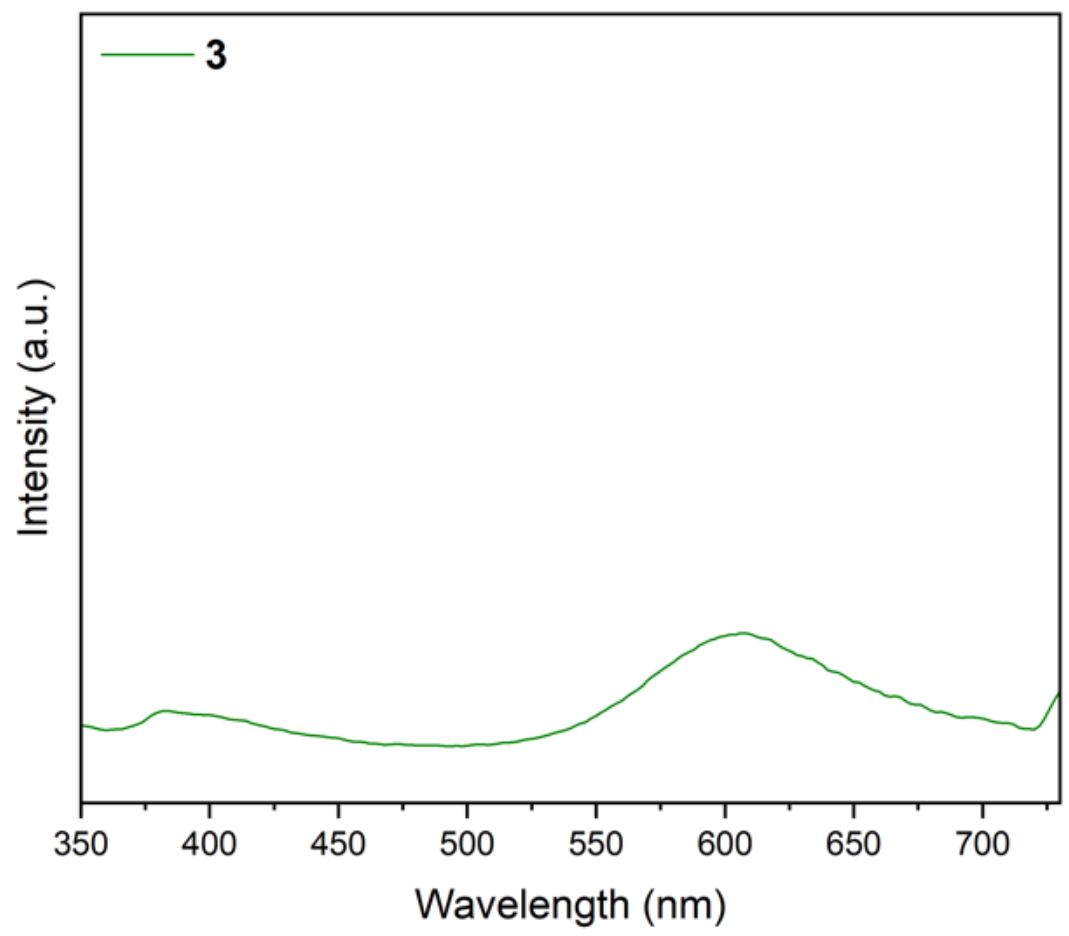

Figure S14. Excitation spectra of ferrocene, [[[(*S*)-(2'-hydroxy-[1,1'-binaphthalen]-2-yl]oxy]carbonyl]- (**3**) monitored at an emission wavelength of 750 nm and recorded without optical filters. a.u. = arbitrary units.

6.4. 2,2'-bipyridine-4,4'-dicarboxylic acid (**4**)

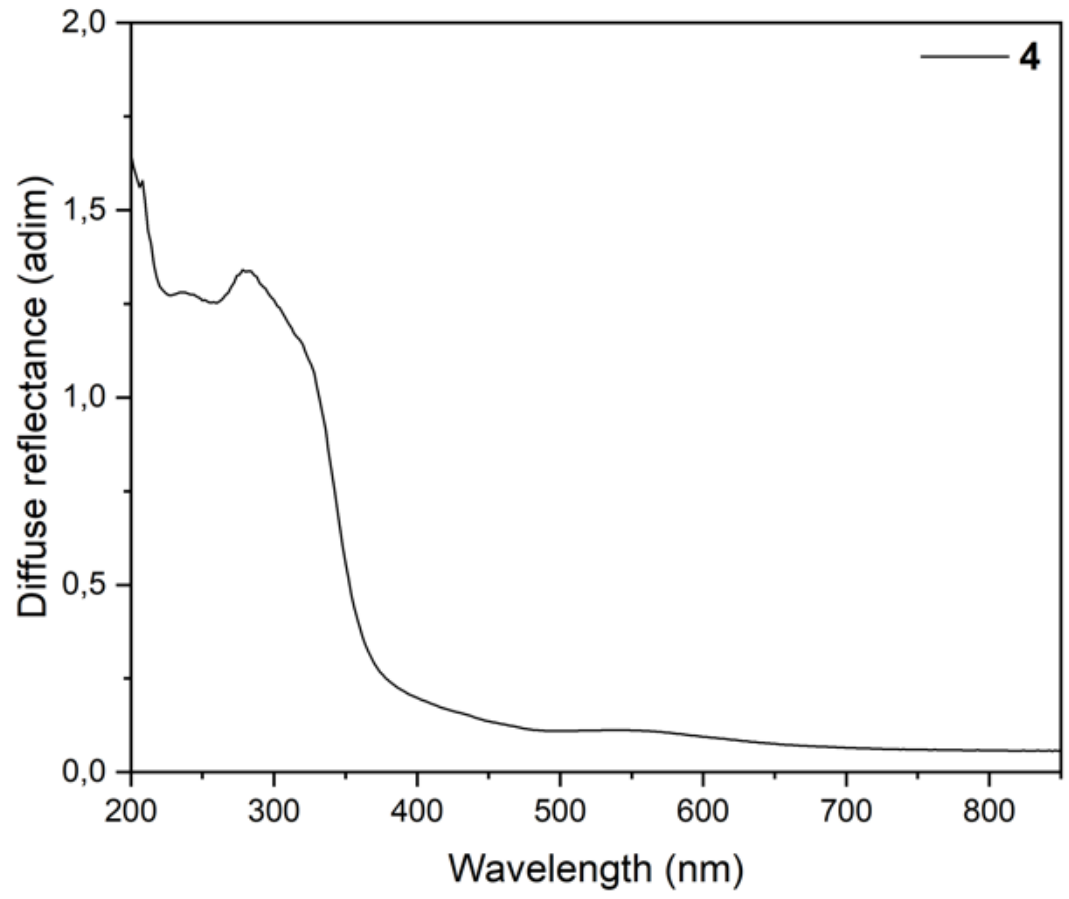

Figure S15. Diffuse reflectance spectra of 2,2'-bipyridine-4,4'-dicarboxylic acid (**4**).

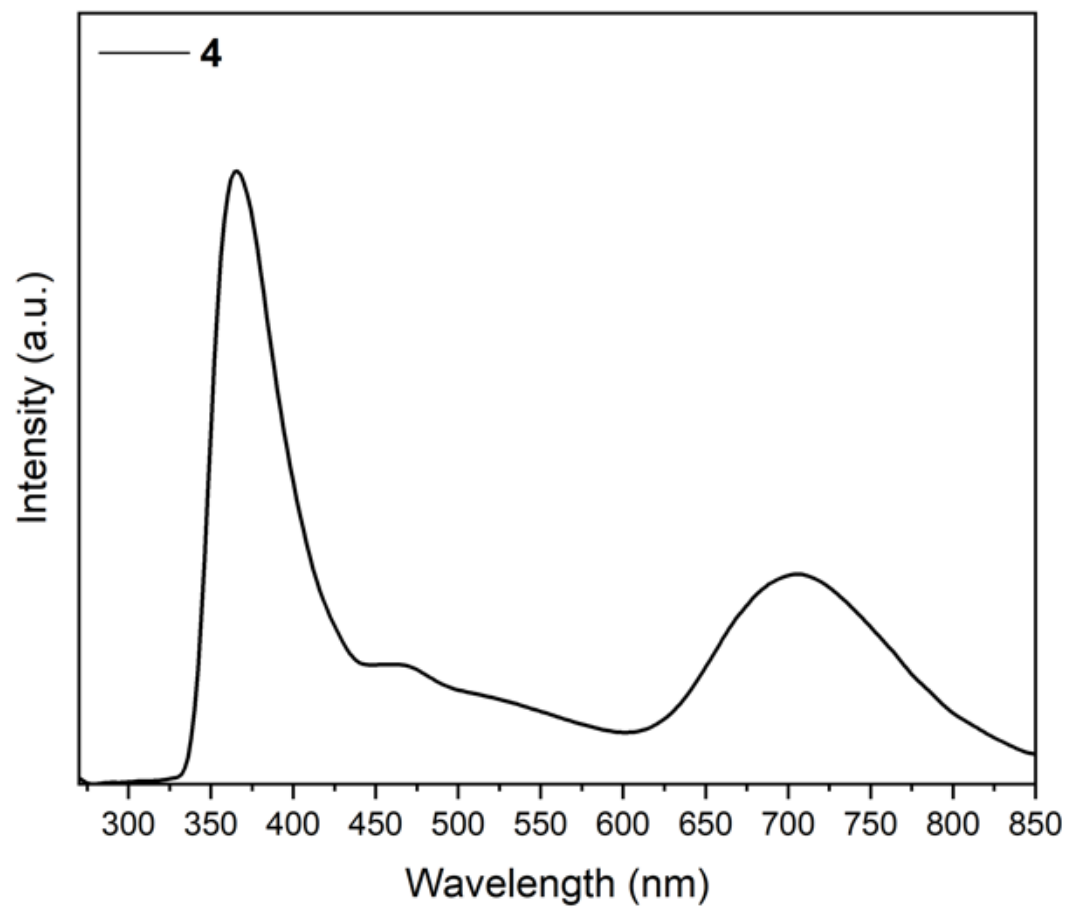

Figure S16. Emission spectra of 2,2'-bipyridine-4,4'-dicarboxylic acid (**4**) recorded upon excitation at 250 nm using a xenon lamp and a 395 nm cutoff filter. a.u. = arbitrary units.

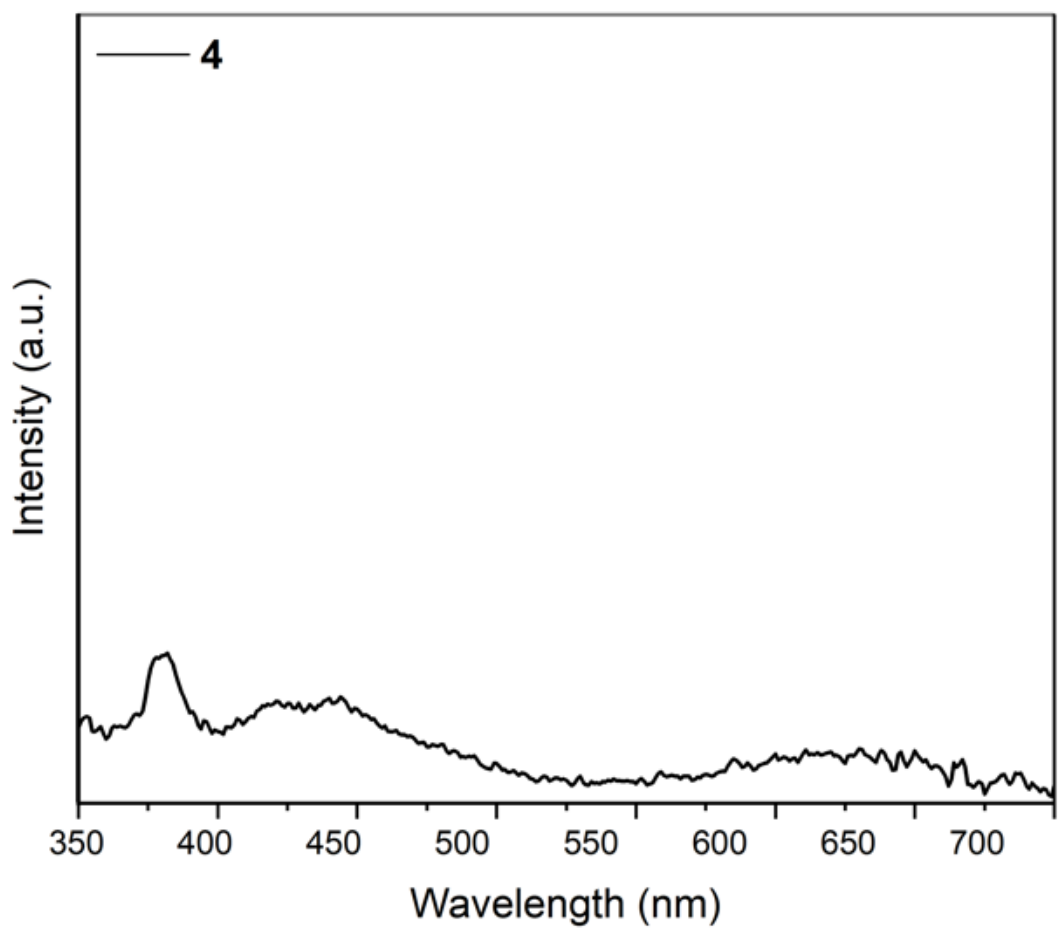

Figure S17. Excitation spectra of 2,2'-bipyridine-4,4'-dicarboxylic acid (**4**) monitored at an emission wavelength of 750 nm and recorded without optical filters. a.u. = arbitrary units.

6.5. Bis((*S*)-2'-((ferrocenecarbonyl)oxy)-[1,1'-binaphthalen]-2-yl) [2,2'-bipyridine]-4,4'-dicarboxylate (**5**)

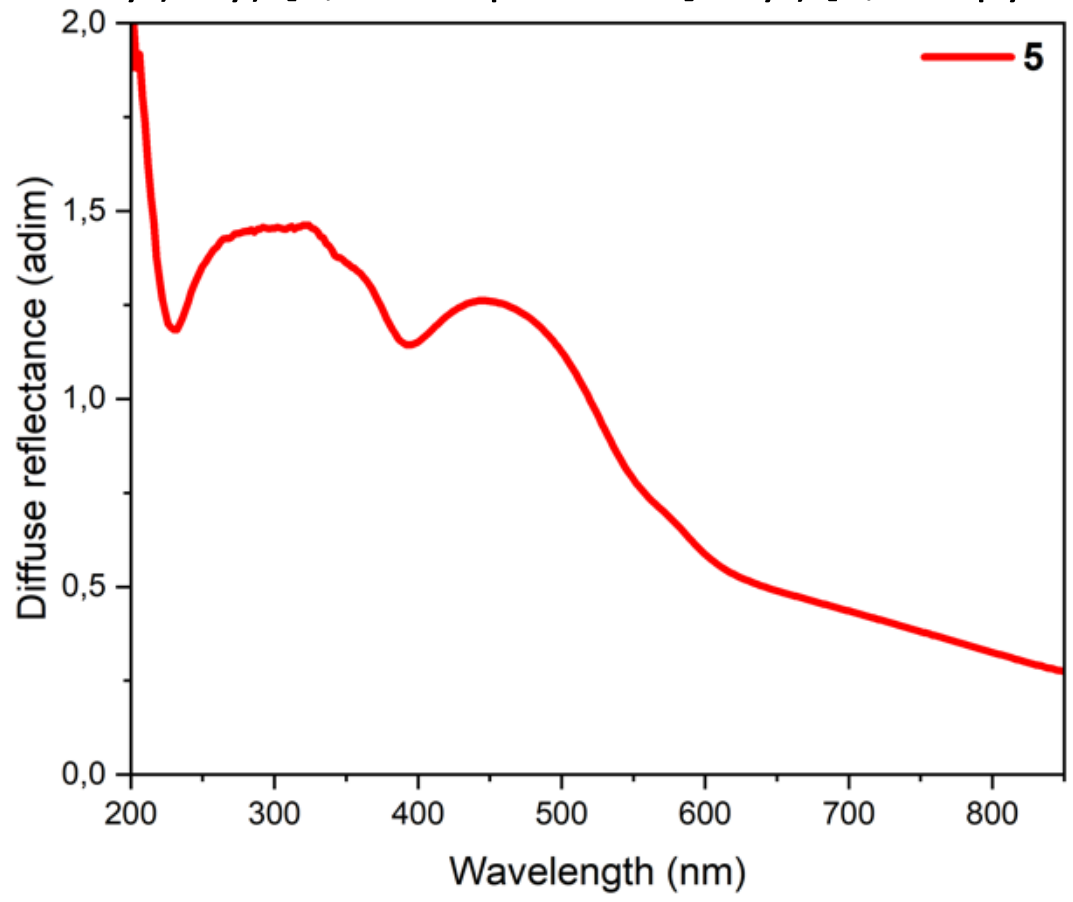

Figure S18. Diffuse reflectance spectra of bis((*S*)-2'-((ferrocenecarbonyl)oxy)-[1,1'-binaphthalen]-2-yl) [2,2'-bipyridine]-4,4'-dicarboxylate (**5**).

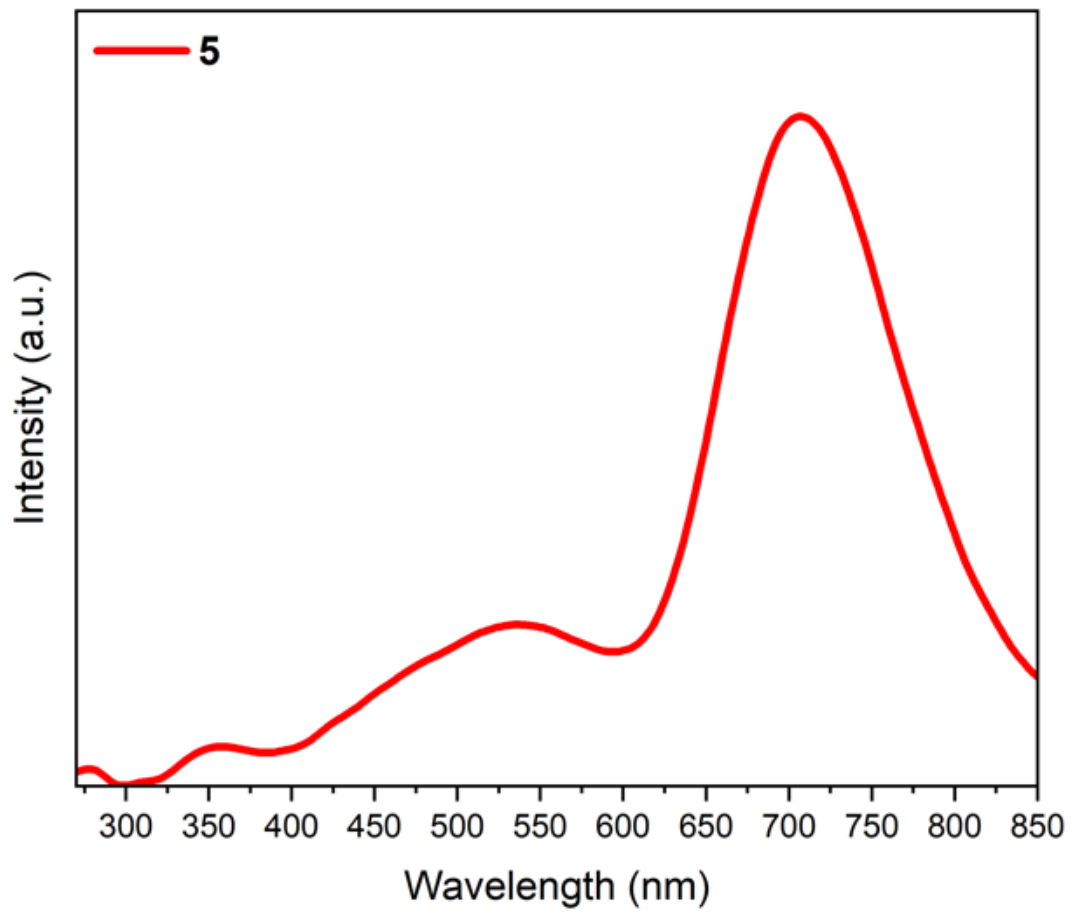

Figure S19. Emission spectra of bis((*S*)-2'-((ferrocenecarbonyl)oxy)-[1,1'-binaphthalen]-2-yl) [2,2'-bipyridine]-4,4'-dicarboxylate (**5**) recorded upon excitation at 250 nm using a xenon lamp and a 395 nm cutoff filter. a.u. = arbitrary units.

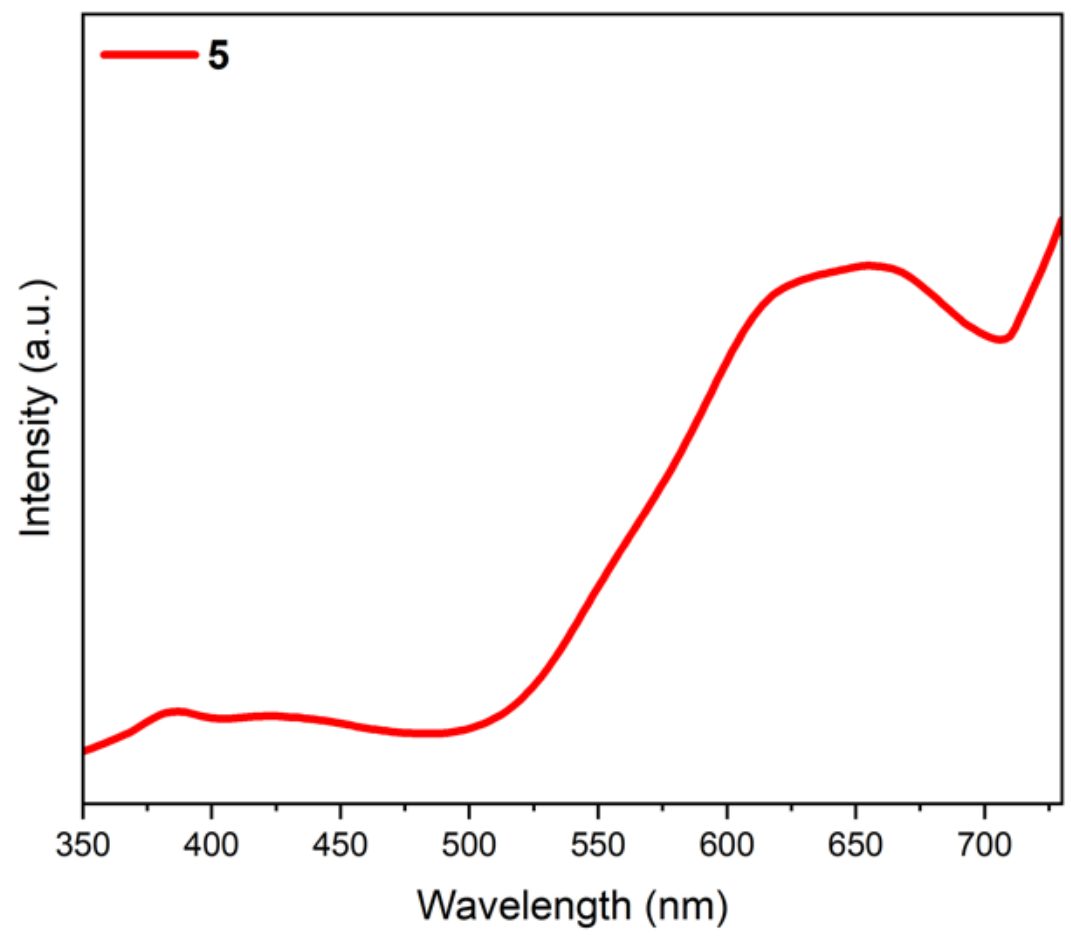

Figure S20. Excitation spectra of bis((*S*)-2'-((ferrocenecarbonyl)oxy)-[1,1'-binaphthalen]-2-yl) [2,2'-bipyridine]-4,4'-dicarboxylate (**5**) monitored at an emission wavelength of 750 nm and recorded without optical filters. a.u. = arbitrary units.

6.6. (*S*)-2'-((ferrocenecarbonyl)oxy)-[1,1'-binaphthalen]-2-yl 4'-(isopropyl(isopropylcarbamoyl)carbamoyl)-[2,2'-bipyridine]-4-carboxylate (**6**)

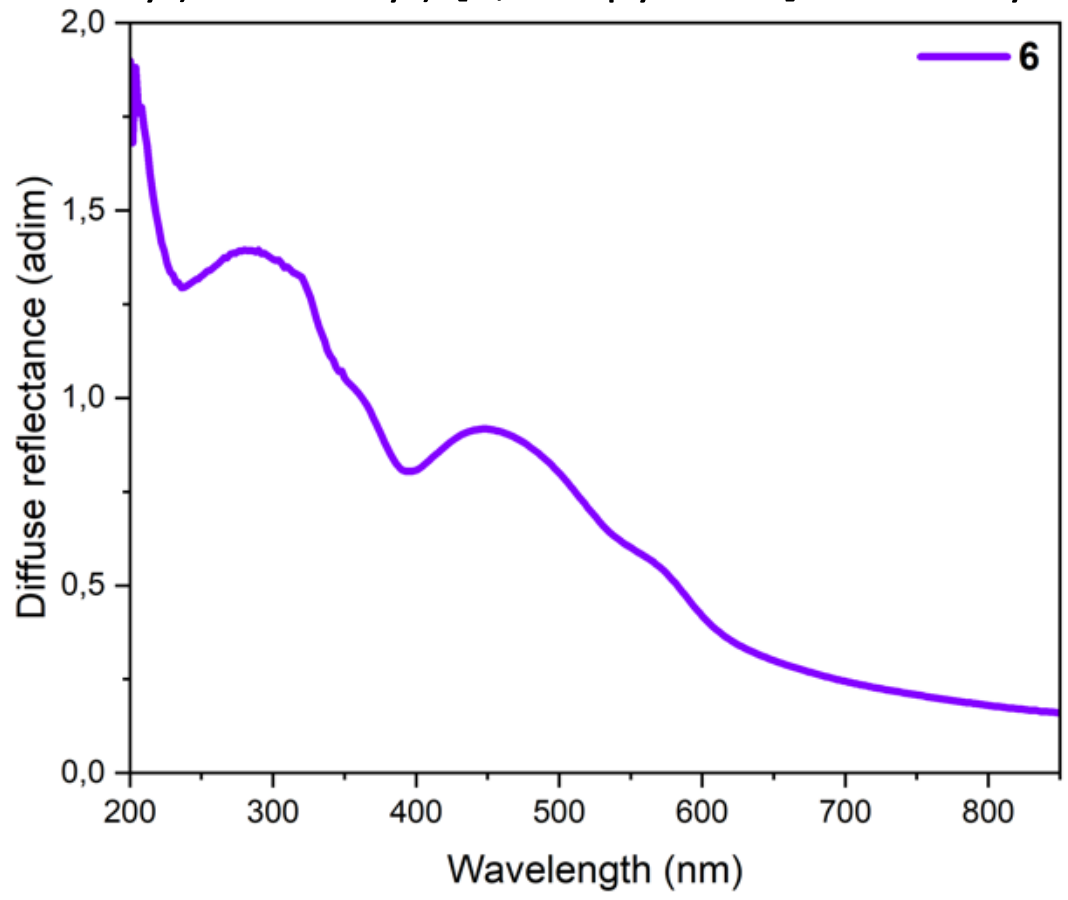

Figure S21. Diffuse reflectance spectra of (*S*)-2'-((ferrocenecarbonyl)oxy)-[1,1'-binaphthalen]-2-yl 4'-(isopropyl(isopropylcarbamoyl)carbamoyl)-[2,2'-bipyridine]-4-carboxylate (**6**).

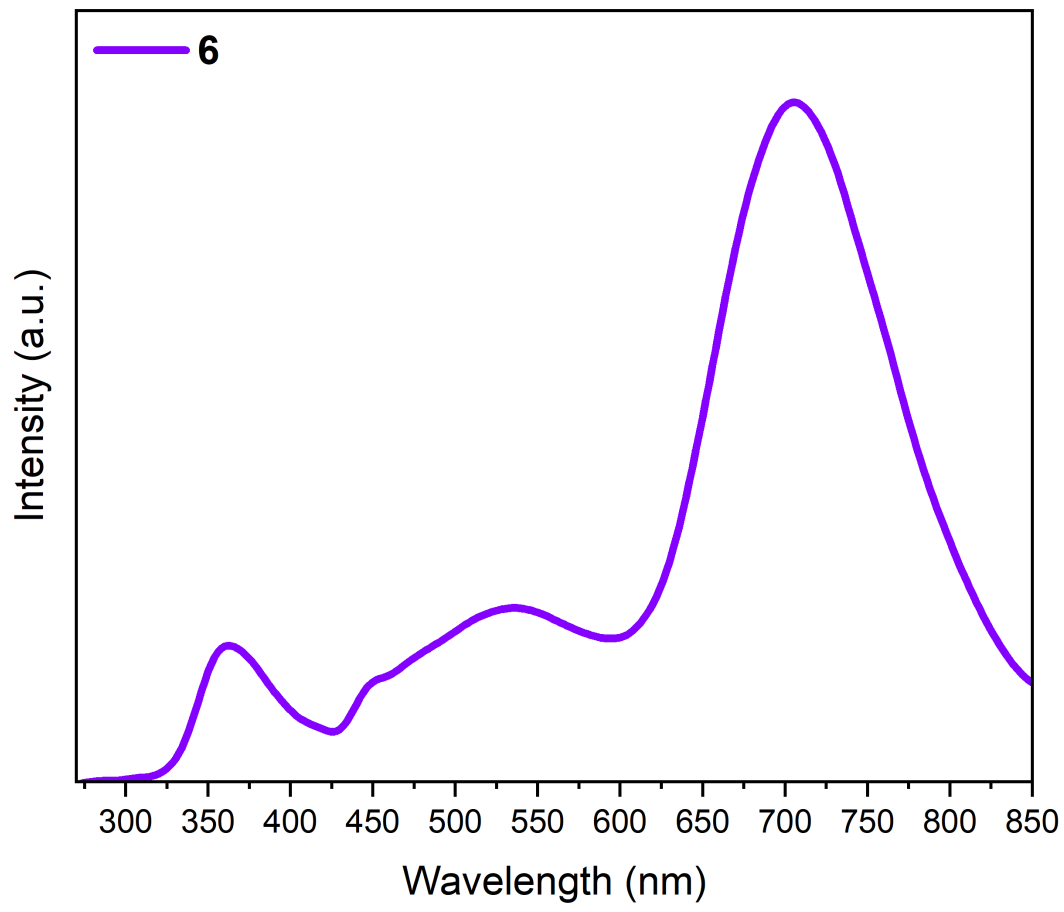

Figure S22. Emission spectra of (*S*)-2'-((ferrocenecarbonyl)oxy)-[1,1'-binaphthalen]-2-yl 4'-(isopropyl(isopropylcarbamoyl)carbamoyl)-[2,2'-bipyridine]-4-carboxylate (**6**) recorded upon excitation at 250 nm using a xenon lamp and a 395 nm cutoff filter. a.u. = arbitrary units.

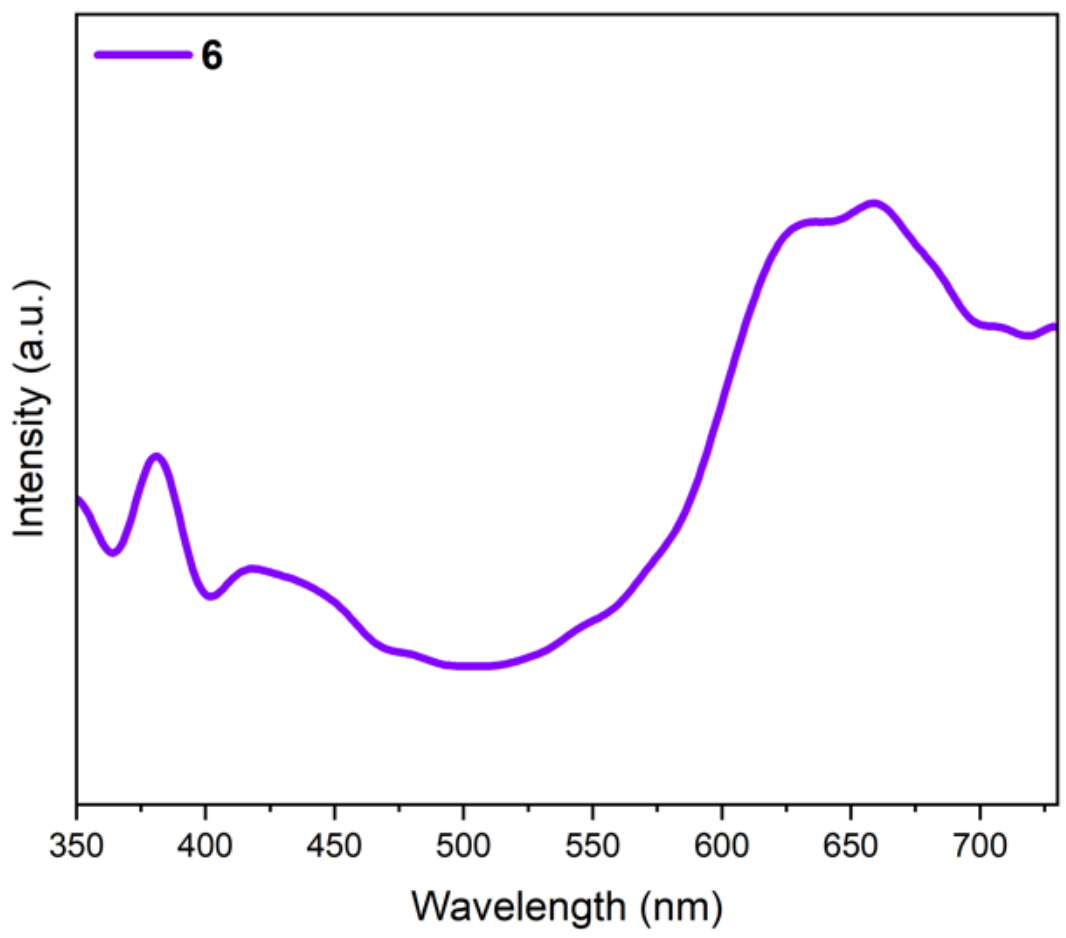

Figure S23. Excitation spectra of (*S*)-2'-((ferrocenecarbonyl)oxy)-[1,1'-binaphthalen]-2-yl 4'-(isopropyl(isopropylcarbamoyl)carbamoyl)-[2,2'-bipyridine]-4-carboxylate (**6**) monitored at an emission wavelength of 750 nm and recorded without optical filters. a.u. = arbitrary units.

6.7. Photographic evidence of visible red emission from **4**

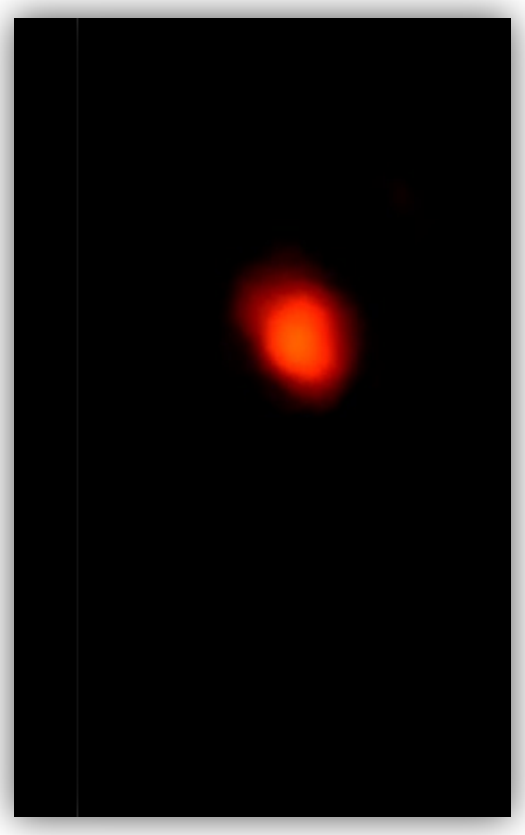

Figure S24. Photographic evidence of the visible red emission from **4**·upon excitation with a 250 nm continuous-wave laser, recorded using a 610 nm long-pass filter.

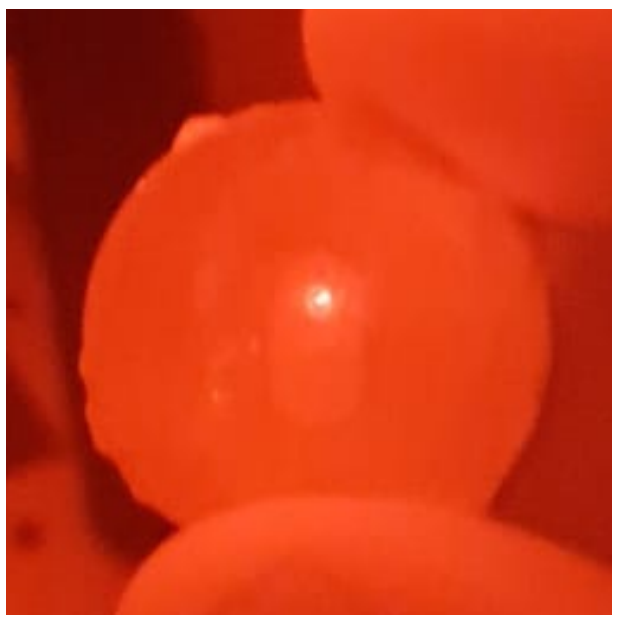

Figure S25. Photographic evidence of the visible red emission from **4**·upon excitation with a 375 nm continuous-wave laser, recorded using a 610 nm long-pass filter.

7. X-Ray diffraction data

Data of benchmark **3** and **5**·EtOAc compounds and **8·$(H_2O)_4$**, were obtained on a Rigaku Oxford Diffraction SuperNova, Dualflex, EosS2, at room temperature, with Mo-K_α radiation (λ = 0.71073 Å). The data were indexed, integrated and scaled with the CrysAlisPro 1.171.43.104a (Rigaku Oxford Diffraction, 2023). The crystal structure was solved by direct methods using the SHELXT 2018/2 software,[4] with the Olex2 1.5 platform. The structures were refined by the full-matrix least-squares technique on F^2 with the SHELXL 2019/3 program.[5] The Flack parameter for benchmarks **3** and **5**·EtOAc were refined to a value of -0.013(18) and -0.020(14) respectively confirming the absolute structure. Other experimental details are given in **Tables S8-S10**. Mercury 2023.3.1 (CCDC, 2001-2013) program was used to visualize and study the hydrogen bonds and another interaction; whereas the Diamond 4.6.8 software was used to generate the structural drawings for this manuscript.[6] Crystallographic data for **3**, **8·$(H_2O)_4$** complex and atropisomer **5**·(EtOAc) have been deposited in the Cambridge Crystallographic Data Center, with the CCDC number CCDC-2535802, 2535803 and 2535804, respectively.

[4] O. V. Dolomanov, L. J. Bourhis, R. J. Gildea, J. A. K. Howard and H. Puschmann, OLEX2: a complete structure solution, refinement and analysis program. *J. Appl. Crystallogr.* 2009, **42**, 339–341.

[5] Sheldrick, G. M. Crystal structure refinement with SHELXL, *Acta Cryst*. 2015, **C71**, 3–8.

[6] (a) Mercury 2023.3.1, Copyright © CCDC, 2001-2023; (b) K Brandenburg, DIAMOND-Crystal and Molecular Structure Visualization Version 4.6.8; Crystal Impact GbR: Bonn, Germany, 2022.

7.1. Crystal information of 3

Table S8. Crystal data and structure refinement for **3**.

Identification CCDC	2535802
Empirical formula	$C_{31}H_{22}FeO_3$
Formula weight	498.363
Temperature/K	293(2)
Crystal system	monoclinic
Space group	$P2_1$
a/Å	7.5288(5)
b/Å	17.0207(12)
c/Å	9.8586(8)
α/°	90
β/°	110.741(8)
γ/°	90
Volume/Å^3	1181.46(16)
Z	2
ρ_{calc}g/cm^3	1.401
μ/mm^{-1}	0.669
F(000)	517.0
Crystal size/mm^3	0.15 × 0.11 × 0.08
Radiation	Mo Kα (λ = 0.71073)
2Θ range for data collection/°	4.78 to 52.74
Index ranges	-9 ≤ h ≤ 9, -21 ≤ k ≤ 17, -12 ≤ l ≤ 12
Reflections collected	7536
Independent reflections	3928 [R_{int} = 0.0413, R_{sigma} = 0.0610]
Data/restraints/parameters	3928/1/317
Goodness-of-fit on F^2	1.044
Final R indexes [I>=2σ (I)]	R_1 = 0.0499, wR_2 = 0.0843
Final R indexes [all data]	R_1 = 0.0659, wR_2 = 0.0910
Largest diff. peak/hole / e Å^{-3}	0.67/-0.34
Flack parameter	-0.013(18)

7.2. Crystal information of 5·EtOAc

Table S9. Crystal data and structure refinement for **5**·EtOAc.

Identification CCDC	2535804
Empirical formula	$C_{78}H_{56}Fe_2N_2O_{10}$
Formula weight	1293.012
Temperature/K	298(2)
Crystal system	Triclinic
Space group	P1
a/Å	9.3471(10)
b/Å	9.5005(9)
c/Å	19.4788(17)
α/°	83.263(8)
β/°	80.067(8)
γ/°	71.758(9)
Volume/Å^3	1614.5(3)
Z	1
ρ_{calc}g/cm^3	1.330
μ/mm^{-1}	0.512
F(000)	671.1
Crystal size/mm^3	0.147 × 0.095 × 0.059
Radiation	Mo Kα (λ = 0.71073)
2Θ range for data collection/°	4.26 to 58.92
Index ranges	-12 ≤ h ≤ 11, -12 ≤ k ≤ 12, -19 ≤ l ≤ 26
Reflections collected	13325
Independent reflections	9358 [R_{int} = 0.0320, R_{sigma} = 0.0584]
Data/restraints/parameters	9358/133/862
Goodness-of-fit on F^2	1.073
Final R indexes [I>=2σ (I)]	R_1 = 0.0594, wR_2 = 0.1224
Final R indexes [all data]	R_1 = 0.0903, wR_2 = 0.1400
Largest diff. peak/hole / e Å^{-3}	0.45/-0.24
Flack and Hooft parameters	-0.020(14) and 0.013(18)

7.3. Crystal information of 8·$(H_2O)_4$

Table S10. Crystal data and structure refinement for **8·$(H_2O)_4$.**

Identification CCDC	2535803
Empirical formula	$C_{52}H_{80}Cl_4Cu_2N_{12}O_{12}$
Formula weight	1334.190
Temperature/K	298.0(5)
Crystal system	Monoclinic
Space group	$P2_1/n$
a/Å	9.8387(2)
b/Å	21.5950(5)
c/Å	15.1262(3)
α/°	90
β/°	96.040(2)
γ/°	90
Volume/Å^3	3195.97(12)
Z	2
ρ_{calc}g/cm^3	1.386
μ/mm^{-1}	0.898
F(000)	1399.5
Crystal size/mm^3	0.18 × 0.14 × 0.12
Radiation	Mo Kα (λ = 0.71073)
2Θ range for data collection/°	3.3 to 56.52
Index ranges	$-12 \le h \le 7$, $-27 \le k \le 28$, $-19 \le l \le 19$
Reflections collected	13214
Independent reflections	6999 [R_{int} = 0.0240, R_{sigma} = 0.0353]
Data/restraints/parameters	6999/0/384
Goodness-of-fit on F^2	1.050
Final R indexes [I>=2σ (I)]	R_1 = 0.0401, wR_2 = 0.0958
Final R indexes [all data]	R_1 = 0.0541, wR_2 = 0.1034
Largest diff. peak/hole / e Å^{-3}	0.96/-0.70
